# *C. elegans* electrotaxis behavior is modulated by heat shock response and unfolded protein response signaling pathways

**DOI:** 10.1038/s41598-021-82466-z

**Published:** 2021-02-04

**Authors:** Shane K. B. Taylor, Muhammad H. Minhas, Justin Tong, P. Ravi Selvaganapathy, Ram K. Mishra, Bhagwati P. Gupta

**Affiliations:** 1 Department of Biology, McMaster University, Hamilton, ON Canada; 2 Department of Mechanical Engineering, McMaster University, Hamilton, ON Canada; 3 Department of Psychiatry and Behavioural Neurosciences, McMaster University, Hamilton, ON Canada

**Keywords:** Behavioural methods, Biological models, Stress and resilience, Behavioural genetics

## Abstract

The nematode *C. elegans* is a leading model to investigate the mechanisms of stress-induced behavioral changes coupled with biochemical mechanisms. Our group has previously characterized *C. elegans* behavior using a microfluidic-based electrotaxis device, and showed that worms display directional motion in the presence of a mild electric field. In this study, we describe the effects of various forms of genetic and environmental stress on the electrotactic movement of animals. Using exposure to chemicals, such as paraquat and tunicamycin, as well as mitochondrial and endoplasmic reticulum (ER) unfolded protein response (UPR) mutants, we demonstrate that chronic stress causes abnormal movement*.* Additionally, we report that *pqe-1* (human RNA exonuclease 1 homolog) is necessary for the maintenance of multiple stress response signaling and electrotaxis behavior of animals. Further, exposure of *C. elegans* to several environmental stress-inducing conditions revealed that while chronic heat and dietary restriction caused electrotaxis speed deficits due to prolonged stress, daily exercise had a beneficial effect on the animals, likely due to improved muscle health and transient activation of UPR. Overall, these data demonstrate that the electrotaxis behavior of worms is susceptible to cytosolic, mitochondrial, and ER stress, and that multiple stress response pathways contribute to its preservation in the face of stressful stimuli.

## Introduction

Organisms have evolved intricate molecular machineries in order to respond to adverse or stressful stimuli as their survival depends on their ability to continuously monitor the environment and mount appropriate responses. Since the initial work of Hans Selye in 1936, who studied stress response in rats and termed it ‘general adaptation syndrome’^1,2^, the work in the field of stress biology has grown exponentially, leading to a better understanding of the connections between stress and diseases. Animals exposed to stressful conditions such as heat, toxins, starvation, and psychological stress activate signaling cascades leading to physiological changes. For instance, in the larvae of fruit fly *Drosophila melanogaster*, noxious stimuli induce a stereotyped escape response mediated by neuronal signaling that manifests as rolling and bending reactions^3^. Work in rats showed that when animals were separated from their mothers as pups they showed increased stress response to environmental conditions^4^. Studies in humans have shown that stress can induce intracellular signaling leading to changes in prefrontal cortex function, thereby impacting learning and memory^5^. In addition, there are numerous reports on the endocrine system responding to stress via signaling cascades mediated by hormones such as adrenaline and cortisol^6^.

Detrimental stress in eukaryotes can often lead to an accumulation of misfolded proteins. The stress caused by this accumulation is mitigated by the molecular machinery consisting of several proteins that constitute the UPR signaling network^7^. The three well-known UPR pathways function in the cytosol, mitochondria, and ER, respectively^7^. The cytosolic heat shock response (HSR), UPR^ER^, and UPR^MT^ regulate both the expression and function of multiple chaperons to maintain homeostasis. These chaperons are members of the heat shock protein family and are transcriptionally regulated by the heat shock factor (HSF), which is a major player in the HSR. Together, UPR^MT^, UPR^ER^, and HSR allay cellular stress by attenuating translation to maintain protein homeostasis within the cell, lowering reactive oxygen species (ROS), and inducing cell death^7^.

UPR^MT^ is activated following mitochondrial dysfunction induced by misfolded protein accumulation within the mitochondria, and disrupts oxidative phosphorylation^8^. The response is similar across eukaryotes, although the regulation in mammals appears to be more complex^8^. Experiments in the nematode *Caenorhabditis elegans* have revealed that ATFS-1 (activating transcription factor associated with stress-1), an ATF5 homolog and bZip transcription factor family member, acts as a sensor protein^9^. ATFS-1 is normally degraded inside healthy mitochondria, but upon mitochondrial dysfunction is transported to the nucleus to regulate gene expression^8^. Failure to reduce mitochondrial stress can result in dysfunctional mitochondria which, if not cleared by the protective mechanism of mitophagy^8,10^, can lead to several deleterious conditions, including premature aging, sarcopenia, and cardiovascular disease^10^.

Similar to mitochondrial stress, ER stress is triggered by the accumulation of misfolded proteins in the ER. The UPR^ER^ response is facilitated by three different transmembrane proteins: inositol-requiring enzyme 1 (IRE1), protein kinase RNA (PKR)-like ER kinase (PERK), and activating transcription factor 6 (ATF6), which then activate distinct signaling pathways. There are significant redundancies between the pathways, with overlapping transcriptional activity. All three pathways regulate the expression of chaperones, including GRP78/BiP^11^. A key transcription factor that modulates UPR^ER^ signaling is the X-Box binding protein-1 (XBP1). While IRE1 is the main regulator of XBP1 activity, ATF6 and PERK also participate in IRE1-XBP1 signaling^11,12^. Failure to regulate ER stress can lead to diseases such as neurodegeneration, metabolic disorders, and cancer^13,14^.

Much of our knowledge of how stress affects behavior and the underlying signaling mechanisms have emerged from studies using a small set of model organisms, including *C. elegans*. This model nematode (worm) is a particularly attractive system due to its genetic tractability, ease of culture, and short life cycle, all of which serve to greatly expedite the rate of discovery^15^. Despite its relative simplicity, approximately half of *C. elegans* genes have homologs in humans and utilize many of the same processes^16^. Cellular stress can affect diverse processes in worms. Manipulations that increase mitochondrial and ER stress affect lifespan, fertility, susceptibility to diseases, and many behavioral processes^17–19^. The small size of the worm greatly facilitates experimentation using microfluidic devices^20^. Our lab has been investigating electrotaxis in *C. elegans* and how it is impacted by stress caused by genetic and environmental factors. In an earlier study, we demonstrated that a mild DC electric field in a microfluidic channel stimulates *C. elegans* to move towards the cathode in a directed manner, a response that is robust, highly repeatable, sensitive, and instantaneous^21^. Electrotactic movement serves as a powerful noninvasive response to evaluate the functional output of the nematode’s locomotory circuit following manipulation^22^. To understand whether the response is affected by treatments that increase stress, we exposed animals to different environmental conditions as well as investigated the impact of genetic mutations. Our findings show that chronic stress compromises electrotaxis speed whereas some forms of transient stress augments it. We found that worms exposed to chemicals that induce ER and mitochondrial stress exhibit reduced speed. A similar phenotype was also observed in mutant strains with disrupted mitochondrial and ER function. The essential role of HSR and UPR^MT^ is further supported by our data showing that animals having defects in both *atfs-1* and *hsf-1* function have a significantly slower movement response. Our analysis of the UPR^ER^ genes reveals that *ire-1/xbp-1* and *pek-1* pathways play essential roles in regulating the electrotaxis of *C. elegans.* We also report that a polyglutamine enhancer-1 protein, PQE-1, maybe involved in the maintenance of HSR and mitochondrial and ER stress, likely due to its role in regulating the global protein synthesis^23,24^. To examine the sensitivity of electrotaxis speed to stress-inducing conditions, we exposed worms to chemical stressors, heat, different bacterial diets, and dietary restriction as well as daily exercise. The results showed that while chronic heat and reduced diet caused a decreased electrotactic movement, exercise had an increased response. Overall, our findings demonstrate that the electrotactic response of *C. elegans* is sensitive to cytosolic, mitochondrial, and ER stress. The results form the basis of future investigations of HSR, UPR^MT^, and UPR^ER^ signaling utilizing microfluidic electrotaxis as a functional output of nematode locomotor circuits.

## Results

### Paraquat-induced oxidative stress reduces the electrotaxis speed of *C. elegans*

**Figure 1:**
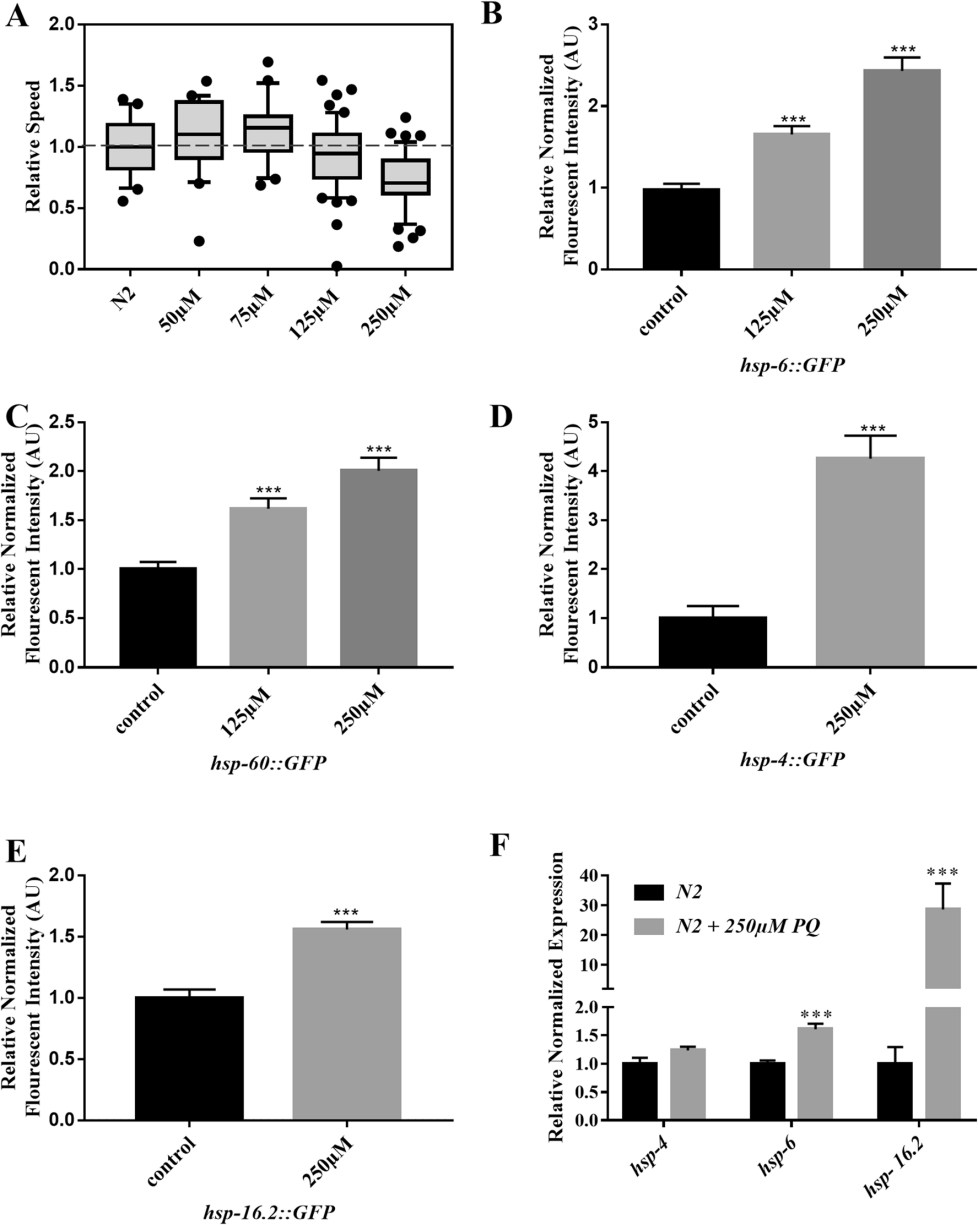
Effect of PQ treatments on electrotaxis and stress response markers. Boxes represent measurements from 25 to 75th percentiles, central horizontal lines represent medians, vertical lines extend to 10th and 90th percentiles, and dots represent outliers. (**A**) Wild-type (N2) animals treated with PQ. PQ did not induce speed abnormalities at 50 μM (*p* = 0.128), 75 μM (*p* = 0.102), or 125 μM (*p* = 0.102), but resulted in speed deficits at 250 μM (*p* < 0.001). (**B, C**) Fluorescence intensity in animals expressing *GFP* under mitochondrial *hsp-6* and *hsp-60* promoters following PQ treatments. GFP fluorescence corresponding to both *hsp-6::GFP* and *hsp-60::GFP* reporters showed increased fluorescence following treatments with 125 μM (*p* < 0.001 in both cases) and 250 μM (*p* < 0.001 and *p* = 0.003 respectively) PQ. (**D, E**) Fluorescence intensity of PQ-treated *hsp-4::GFP* and *hsp-16.2::GFP* animals. Fluorescence was increased following treatment with 250 μM PQ (*p* < 0.001 in both cases). (**F**) Quantitative reverse transcription-polymerase chain reaction (RT-qPCR) analysis showed an increase in *hsp-6* (*p* < 0.0001) and *hsp-16.2* (*p* = 0.0007) but not *hsp-4* (*p* = 0.1125) transcripts in N2 day-1 adults following exposure to 250 μM PQ. Number of animals were (**A**) N2 untreated: n = 80, N2 + 50 μM PQ: n = 20, N2 + 75 μM PQ: n = 20, N2 + 125 μM PQ: n = 50, N2 + 250 μM PQ: n = 45. (**B**) *hsp-6::GFP* untreated: n = 40, *hsp-6::GFP* + 125 μM PQ: n = 32, *hsp-6::GFP* + 250 μM PQ: n = 31, (**C**) *hsp-60::GFP* untreated: n = 33, *hsp-60::GFP* + 125 μM PQ: n = 34, *hsp-60::GFP* + 250 μM PQ: n = 35. (**D**) *hsp-4::GFP* untreated: n = 27, *hsp-4::GFP* + 250 μM PQ: n = 29, (**E**) *hsp-16.2::GFP* untreated: n = 31, *hsp-16.2::GFP* + 250 μM PQ: n = 20. (**F**) N2 untreated: n = 2 batches, N2 + 250 μM PQ: n = 2 batches. Statistical analyses for panels A-E were done using one-way ANOVA with Dunnett’s post hoc test. qPCR results in F were generated by BioRad’s CFX Maestro software (version 3.1, https://www.bio-rad.com/en-ca/category/qpcr-analysis-software) and data was analyzed using one-way ANOVA with Tukey’s post hoc test. AU: Arbitrary Unit.

Oxidative stress is known to affect the behavioral responses of *C. elegans*^17,18,25^. To investigate whether this form of stress can also alter electrotaxis in animals, we used paraquat (PQ), a herbicide that disrupts mitochondrial function by inducing the generation of superoxides^26,27^. High concentrations of PQ have a detrimental effect on the lifespan of worms and cause damage to dopaminergic (DA) and other neurons by increasing ROS levels^17,18,26–28^. We examined the effect of PQ-induced stress on electrotaxis by exposing animals to different doses of the chemical. While exposures to low or moderate concentrations ranging from 50 to 125 µM had no effect on the movement of day-1 adults, 250 µM of PQ caused a significant defect, resulting in a slower speed in the microfluidic channel (Fig. 1A). In addition to electrotaxis, PQ-exposed animals showed increased expression of mitochondrial chaperones. Specifically, the mitochondrial stress response reporters *hsp-6::GFP* (HSP70 family) and *hsp-60::GFP* (HSP60 family) were significantly upregulated (Fig. 1B,C).

To determine whether PQ affects other processes, we examined the expression of two other chaperones for ER and cytosolic stress, namely HSP-4 (BiP/GRP78 family member, ER stress) and HSP-16.2 (HSP16 family, cytosolic stress) following PQ treatment. Although the GFP reporters for both these chaperons showed a statistically significant increase in fluorescence, the fold change of the ER GFP reporter was much higher than that of mitochondrial reporters (Fig. 1D,E). Similar to changes in fluorescence reporters, the transcript analysis also showed higher levels of *hsp-6* and *hsp-16.2* following PQ exposure (Fig. 1F). We did not see a significant increase in *hsp-4* transcript. These results are consistent with the previously reported finding that PQ induces both mitochondrial and ER stress in different animal models^29–31^. Overall, PQ causes multiple stresses in *C. elegans* that contribute to a decrease in electrotaxis speed.

### Mutations in genes regulating mitochondrial function affect electrotaxis

To further investigate whether the electrotactic response is perturbed by mitochondrial dysfunction, we conducted a survey of mutants affecting mitochondrial processes and UPR^MT^. The set of genes consisted of an ortholog of ubiquinol-cytochrome C reductase (*isp-1*, Rieske iron sulfur protein subunit of mitochondrial complex III), a subunit of mitochondrial complex I (*gas-1*), a subunit of mitochondrial complex II (*mev-1*, cytochrome b), PTEN-induced kinase 1 (*pink-1*), mitochondrial uncoupling protein (UCP) ortholog (*ucp-4*), two mitochondrial superoxide dismutase orthologs (*sod-2* and *sod-3*), coenzyme Q7 ortholog (*clk-1*), and *atfs-1*. Some of these genes are reported to affect motility of *C. elegans* on solid media and thrashing in liquid environment^32–35^.

**Figure 2:**
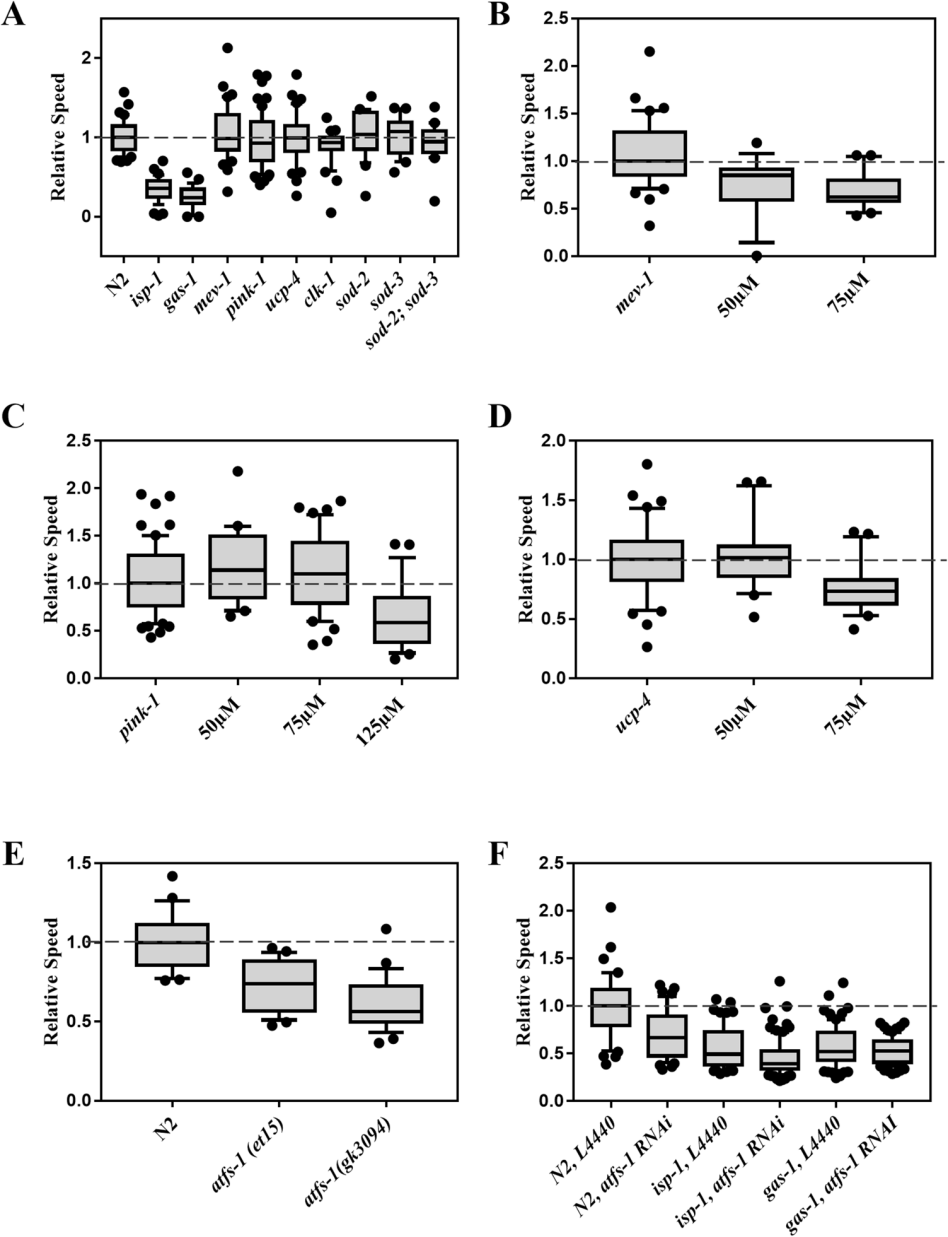
Electrotaxis of mitochondrial mutants. Refer to Fig. 1 for a description of box plot. (**A**) *isp-1(qm150)* (*p* < 0.001) and *gas-1(fc21)* (*p* < 0.001) animals exhibit speed defects. In contrast, *mev-1(kn1)* (*p* = 0.567), *pink-1(ok3538)* (*p* = 0.239), *ucp-4(ok195)* (*p* = 0.942), *clk-1(e2519)* (*p* = 0.065), *sod-2(gk257)* (*p* = 0.509), *sod-3(tm760)* (*p* = 0.578), and *sod-2(gk257); sod-3(tm760)* double (*p* = 0.485) mutants are comparable to the wild-type (N2) control. (**B**) *mev-1(kn1)* animals exhibited significant slowness following treatment with 50 μM (*p* = 0.003) or 75 μM (*p* < 0.001) PQ. (**C**) *pink-1(ok3538)* animals showed no obvious defect following treatment with 50 μM (*p* = 0.394) or 75 μM (*p* = 0.694) PQ, but were significantly slower following treatment with 125 μM PQ (*p* < 0.001). (**D**) *ucp-4(ok195)* animals were normal following treatment with 50 μM (*p* = 0.946), but showed significant slowness when treated with 75 μM PQ (*p* = 0.002). (**E**) *atfs-1(et15)* and *atfs-1(gk3094)* animals showed defective electrotaxis phenotype (*p* < 0.001 and *p* = 0.008, respectively). (**F**) *atfs-1* RNAi resulted in a significant decrease in the electrotaxis speed of N2 (*p* < 0.001) and *isp-1(qm150)* (*p* = 0.0480) animals whereas it had no effect on the speed of *gas-1(fc21)* (*p* = 0.7929) animals. The numbers of animals were (**A**) N2: n = 40, *isp-1(qm150)*: n = 39, *gas-1(fc21)*: n = 29, *mev-1(kn1)*: n = 40, *pink-1(ok3538)*: n = 61, *ucp-4(ok195)*: n = 40, *clk-1(e2519)*: n = 30, *sod-2(gk257)*: n = 21, *sod-3(tm760)*: n = 20, *sod-2(gk257); sod-3(tm760)*: n = 20. (**B**) N2: n = 29, *atfs-1(et15)*: n = 26, *atfs-1(gk3094)*: n = 26. (**C**) *mev-1*(*kn1*) untreated: n = 20, + 50 μM PQ: n = 18, + 75 μM PQ: n = 20. (**D**) *pink-1*(*ok3538*) untreated: n = 33, + 50 μM PQ: n = 20, + 75 μM PQ: n = 40, + 125 μM PQ: n = 27. (**E**) *ucp-4*(*ok195*) untreated: n = 20, + 50 μM PQ: n = 20, + 75 μM PQ: n = 20. (**F**) N2, L4440: n = 45**,** N2, *atfs-1* RNAi: n = 44, *isp-1(qm150),* L4440: n = 60, *isp-1(qm150), atfs-1* RNAi: n = 93, *gas-1(fc21),* L4440: n = 76, *gas-1(fc21) atfs-1* RNAi: n = 71. In all cases statistical analyses were done using one-way ANOVA with Dunnett’s post hoc test.

Analysis of the electrotactic response showed that most of the mutant animals, including *mev-1(kn1)*, *pink-1(ok3538)*, *ucp-4(ok195)*, *clk-1(e2519)*, *sod-2(gk257)*, *sod-3(tm760)*, and *sod-2(gk257); sod-3(tm760)* double, had no obvious defects; however, two particular mutants, *isp-1(qm150)* and *gas-1(fc21)*, showed a significantly reduced electrotaxis speed (Fig. 2A). Although both genes encode components of the electron transport chain, *isp-1(qm150)* is long-lived with resistance to oxidative damage^36^, while *gas-1(fc21)* is short-lived with increased ROS and sensitivity to oxidative damage^37^, suggesting that neither lifespan differences nor ROS toxicity correlate with electrotaxis phenotypes. This notion is supported by the other mutant data, indicating that *mev-1(kn1)* has increased ROS and oxidative damage with short lifespan, *clk-1(e2519)* shows decreased oxidative damage and long lifespan, and *sod* mutants show increased oxidative damage with normal or extended lifespans, yet all exhibit similar electrotaxis speeds (Fig. 2A)^38,39^.

We chose a subset of mitochondrial genes for further investigation. Specifically, we explored the possibility that mutant animals may not exhibit an electrotaxis phenotype on their own, but may do so when subjected to further stress. Consistent with this, *mev-1(kn1)* and *pink-1(ok3538)*, which show sensitivity to PQ^18,38^, displayed electrotaxis defects at exposure concentrations that do not alter the speed of control animals. Specifically, *mev-1(kn1)* animals exhibited speed deficits when cultured under chronic exposures to either 50 μM or 75 μM PQ (Fig. 2B). Similarly, *pink-1(ok3538)* animals were also affected, displaying significantly reduced electrotaxis speed at 125 μM PQ (Fig. 2C). Interestingly, *ucp-4(ok195)* adults displayed increased sensitivity to PQ under our chronic exposure paradigm, exhibiting slow electrotaxis at 75 μM (Fig. 2D). Thus, PQ-induced stress and mitochondrial defects perturb *C. elegans* electrotaxis speed.

To further investigate the effect of perturbations in UPR^MT^ on electrotaxis, we examined the phenotypes of two different *atfs-1* alleles, a genetic null *atfs-1(gk3094)* and a gain-of-function *atfs-1(et15).* While the *gk3094* mutation prevents the UPR^MT^ response, an opposite phenotype is caused by *et15*^40–42^. Additionally, the HSR is activated in *gk3094* animals^41^. Both *atfs-1* mutants displayed significantly slower speeds than wild-type controls, although the null allele phenotype was most severe (Fig. 2E). We also knocked down *atfs-1* in *isp-1* and *gas-1* mutant strains and found that while the electrotaxis speed of *isp-1(qm150)* was reduced, there was no change in *gas-1(fc21)* animals (Fig. 2F). Collectively, these data demonstrate that electrotaxis speed is sensitive to disruptions in mitochondrial function, UPR^MT^, and HSR.

### Impairment of UPR^ER^ causes electrotaxis speed deficits

**Figure 3:**
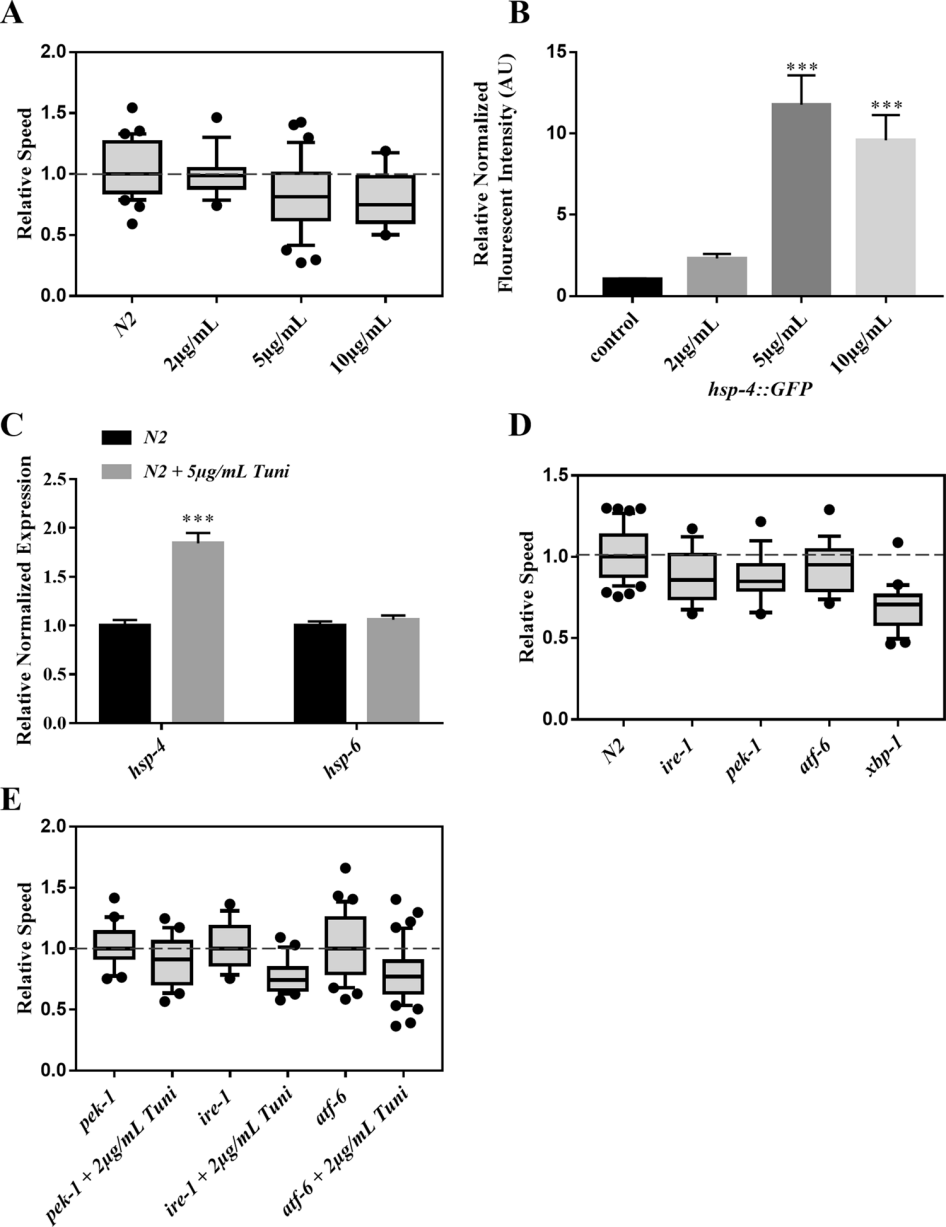
Electrotaxis phenotype of tunicamycin-treated animals and UPR^ER^ mutants. Refer to Fig. 1 for a description of box plot. (**A**) Wild-type (N2) animals show no speed abnormalities when exposed to 2 μg/mL of tunicamycin (*p* = 0.590), but exhibit speed deficits at higher doses, 5 μg/mL (*p* = 0.002) and 10 μg/mL (*p* = 0.008). (**B**) Fluorescence intensity of tunicamycin-treated *hsp-4::GFP* animals. The fluorescence was significantly increased following treatments with 5 μg/mL, and 10 μg/mL tunicamycin (*p* < 0.001). **C**) RT-qPCR analysis of chaperons *hsp-4* (*p* = 0.003) and *hsp-6* (*p* = 0.3652) in N2 day-1 adults following exposure to 5 μg/mL of Tunicamycin. (**D**) Electrotaxis speed of N2 and UPR^ER^ mutants. While *ire-1(v33)* (*p* = 0.0144)*, pek-1(ok275)* (*p* = 0.0082) *and xbp-1(zc12)* (*p* = 0.0001) animals exhibited significant speed defects, *atf-6(ok551)* mutants were not significantly affected (*p* = 0.2995). (**E**) Responses of mutants following 2 μg/mL tunicamycin treatments., Animals showed a significant decline in the speed. P values are 0.0333 for *pek-1(ok275),* < 0.0001 for *ire-1(v33),* and 0.0004 for *atf-6(ok551)*. The numbers of animals were: (**A**) N2 untreated: n = 30, N2 + 2 μg/mL Tuni: n = 15, N2 + 5 μg/mL Tuni: n = 32, N2 + 10 μg/mL Tuni: n = 10. (**B**) *hsp-4::GFP* untreated: n = 45, *hsp-4::GFP* + 2 μg/mL Tuni : n = 32, *hsp-4::GFP* + 5 μg/mL Tuni: n = 37, *hsp-4::GFP* + 10 μg/mL Tuni: n = 31. (**C**) N2 untreated: n = 3 batches, N2 + 5 μg/mL Tuni: n = 3 batches. (**D**) N2: n = 42, *ire-1*: n = 17, *pek-1*: n = 18, *atf-6*: n = 18, *xbp-1*: n = 23. (**E**) *pek-1:* n = *18, pek-1* + 2 μg/mL Tuni: n = 20, *ire-1:* n = *17, ire-1* + 2 μg/mL Tuni: n = 21, *atf-6:* n = *33, atf-6* + 2 μg/mL Tuni: n = 44. One-way ANOVA was performed followed by Dunnett’s post hoc test for A and B, which showed that all conditions except one (i.e., control vs. 2 µg in panel B, *p* = 0.7410) were significant. Student’s unpaired t-test was used for the remaining drug conditions. Data in C was analyzed using one-way ANOVA with Tukey’s post hoc test.

Our finding that PQ induces the expression of ER chaperone *hsp-4::GFP* (Fig. 1C) led us to investigate the impact of ER stress on electrotaxis using tunicamycin, a chemical that promotes protein misfolding by inhibiting protein glycosylation^43,44^. As shown in Fig. 3A, chronic exposure to tunicamycin at concentrations of 5 μg/mL or greater significantly reduced electrotaxis speed. These results are supported by the analysis of *hsp-4::GFP* reporter expression and *hsp-4* transcript (Fig. 3B,C), suggesting the detrimental effect of tunicamycin exposure^40^. As expected, *hsp-6* level was unchanged (Fig. 3C). We did not test electrotactic responses of animals at concentrations higher than 10 μg/mL due to significant toxicity, as judged by the highly pronounced larval lethality and slow growth of escapers at this concentration (data not shown).

Our group had previously demonstrated the role of dopaminergic neurons in mediating electrotaxis^22^. Therefore, we examined whether tunicamycin-induced ER stress will affect these neurons in our assay. The analysis of *dat-1::*YFP reporter revealed visible defects in neuronal morphology in treated animals illustrated by dendritic/axonal abnormalities and loss of cell bodies (Fig. S1A). A similar result was obtained following PQ treatment (Fig. S1B). These data suggest that toxin-induced neuronal damage may contribute to electrotaxis deficits in animals.

We further characterized ER disruption by testing the phenotypes of UPR^ER^ pathway mutants, specifically *ire-1*, *pek-1, atf-6,* and *xbp-1*. The speed was significantly lower in *ire-1(v33), xbp-1(zc12),* and *pek-1(ok275) but not atf-6(ok551)* animals compared to the control, (Fig. 3D). Interestingly, the defect was most pronounced in *xbp-1* mutants, which may be due to the gene being regulated by multiple UPR^ER^ arms. We also examined the sensitivity of UPR^ER^ mutants by exposing them to tunicamycin. Treatment with 2 μg/mL caused a significant decrease in the mobility of all three strains (Fig. 3E). A higher concentration (5 μg/mL) was found to be toxic to worms, resulting in lethality (data not shown). Taken together, the results show that electrotaxis is susceptible to ER stress-inducing conditions, and all three UPR^ER^ transducers function to facilitate this behavior.

**Figure 4:**
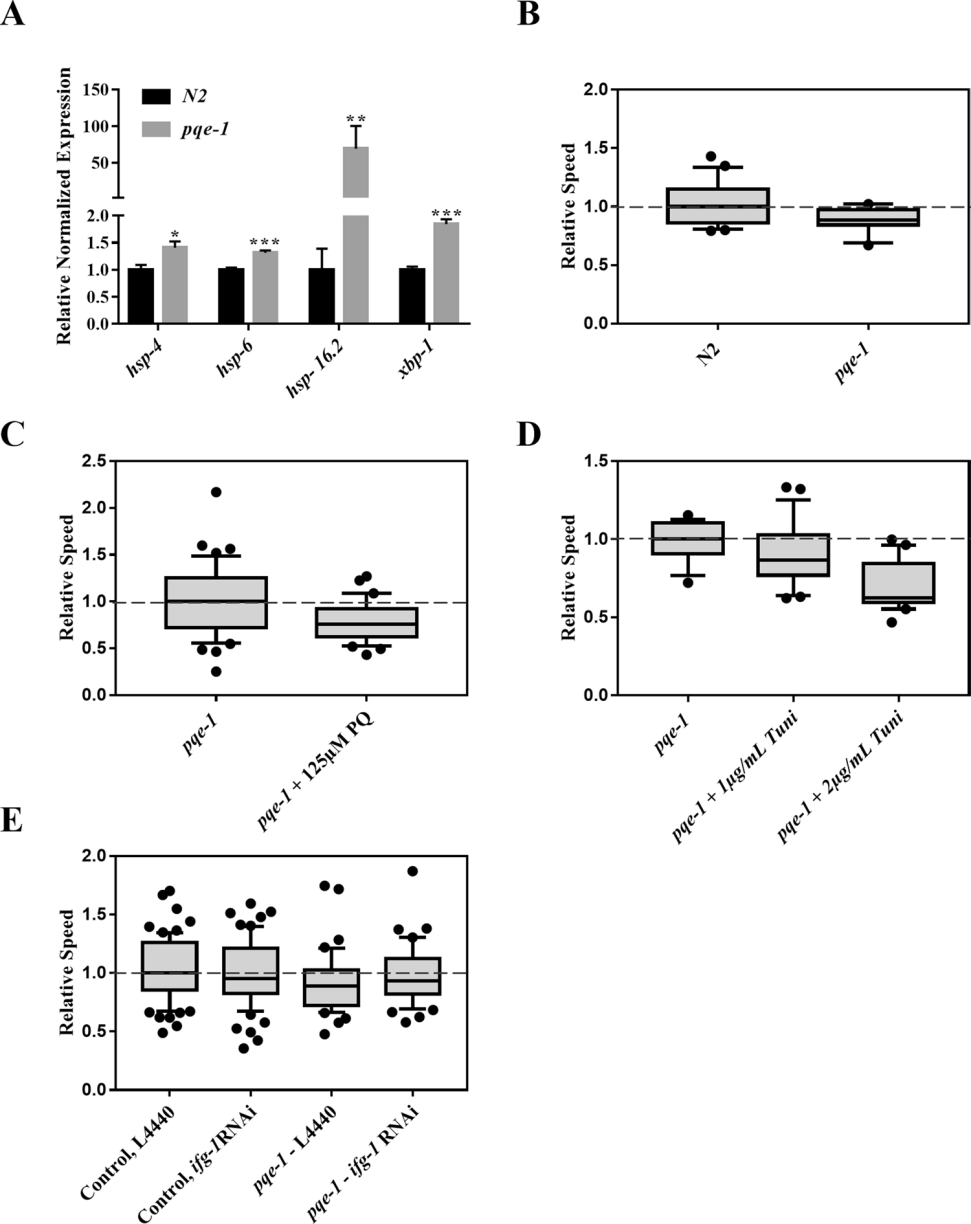
Analysis of *pqe-1* mutants under different stress-inducing conditions. Refer to Fig. 1 for a description of box plot. (**A**) RT-qPCR analysis showing increased expression of *hsp-4* (*p* = 0.0128), *hsp-6* (*p* < 0.0001), *hsp-16.2* (*p* = 0.0012) and *xbp-1*(*p* < 0.0001) in *pqe-1*(ok1983) day-1 adults. (**B**) *pqe-1(ok1983)* animals exhibited a reduced electrotaxis speed compared to wild-type N2 (*p* = 0.0053) (**C**) The speed was further decreased following treatments with 125 μM PQ (*p* = 0.007). (**D**) The speed of *pqe-1(ok1983)* was not significantly different from untreated control (*p* = 0.2461). At 2 μg/mL exposure, *pqe-1* mutants showed a significant decrease in speed (*p* = 0.0001). **(E**) The electrotaxis speed of *ifg-1 RNAi* in control and *pqe-1(ok1983)* animals. The *ifg-1* RNAi had no effect on control animals (*p* = 0.4193) or *pqe-1* mutants (*p* = 0.6676). The numbers of animals were (**B**) N2: n = 3 batches, *pqe-1* n = 3 batches. (**C**) *pqe-1* untreated: n = 44, *pqe-1* + PQ 125 μM: n = 30. (**D**) *pqe-1: n* = *16, pqe-1* + 1 μg/mL Tuni n = 21, *pqe-1* + 2 μg/mL Tuni n = 21. (**E**) Control, L4440: n = 47, Control, *ifg-1* RNAi: n = 66, *pqe-1,* L4440: n = 41, *pqe-1, ifg-1* RNAi: n = 44. Data was analyzed using one-way ANOVA with Tukey’s post hoc test (A), one-way ANOVA with Dunnett’s post hoc test (D), and unpaired Student’s t-test (B,C,E).

Next, the role of *pqe-1* was investigated based on its potential link to ER stress. Earlier, *pqe-1* mutants were reported to cause an increase in transgene expression and enhance polyglutamine (poly-Q) neurotoxicity^23,24^. We speculated that both these phenotypes may be caused by proteotoxic stress due to a failure to regulate global protein synthesis. In support of this, *pqe-1(ok1983)* animals showed an increase in the expression of *hsp-4* chaperon and *xbp-1* (Fig. 4A, Fig. S2A). Interestingly, *hsp-6* and *hsp16.2* levels were also upregulated (Fig. 4A). As expected, the electrotaxis speed of mutant animals was significantly reduced (Fig. 4B), and the phenotype was exacerbated by treatment with PQ and tunicamycin (Fig. 4C,D). The sensitivity towards tunicamycin was particularly pronounced; although wild-type animals showed no abnormal electrotaxis phenotype at 2 μg/mL tunicamycin, *pqe-1* mutants exhibited a significantly reduced speed (compare Fig. 4D with 3A). Concentrations of tunicamycin above 2 μg/mL induced growth arrest and lethality in *pqe-1(ok1983)* worms (data not shown). The mutant animals also exhibited an increased degeneration of dopaminergic neurons and shorter lifespan (Fig. S2B,C).

We investigated whether reducing protein translation would lower proteotoxicity leading to a rescue of the electrotaxis defect of *pqe-1* mutant animals. This was tested by knocking down the eIF4G homolog, *ifg-1*, that encodes a component of the eIF4 complex^45^. The results showed that *ifg-1* RNAi was unable to suppress the phenotype of *pqe-1* mutant (Figs. 4E, S3). Overall, these observations provide the first evidence that *pqe-1* plays an essential role in maintaining multiple stress response pathways and electrotactic response of *C. elegans*.

### Chronic heat impairs the electrotactic response

**Figure 5:**
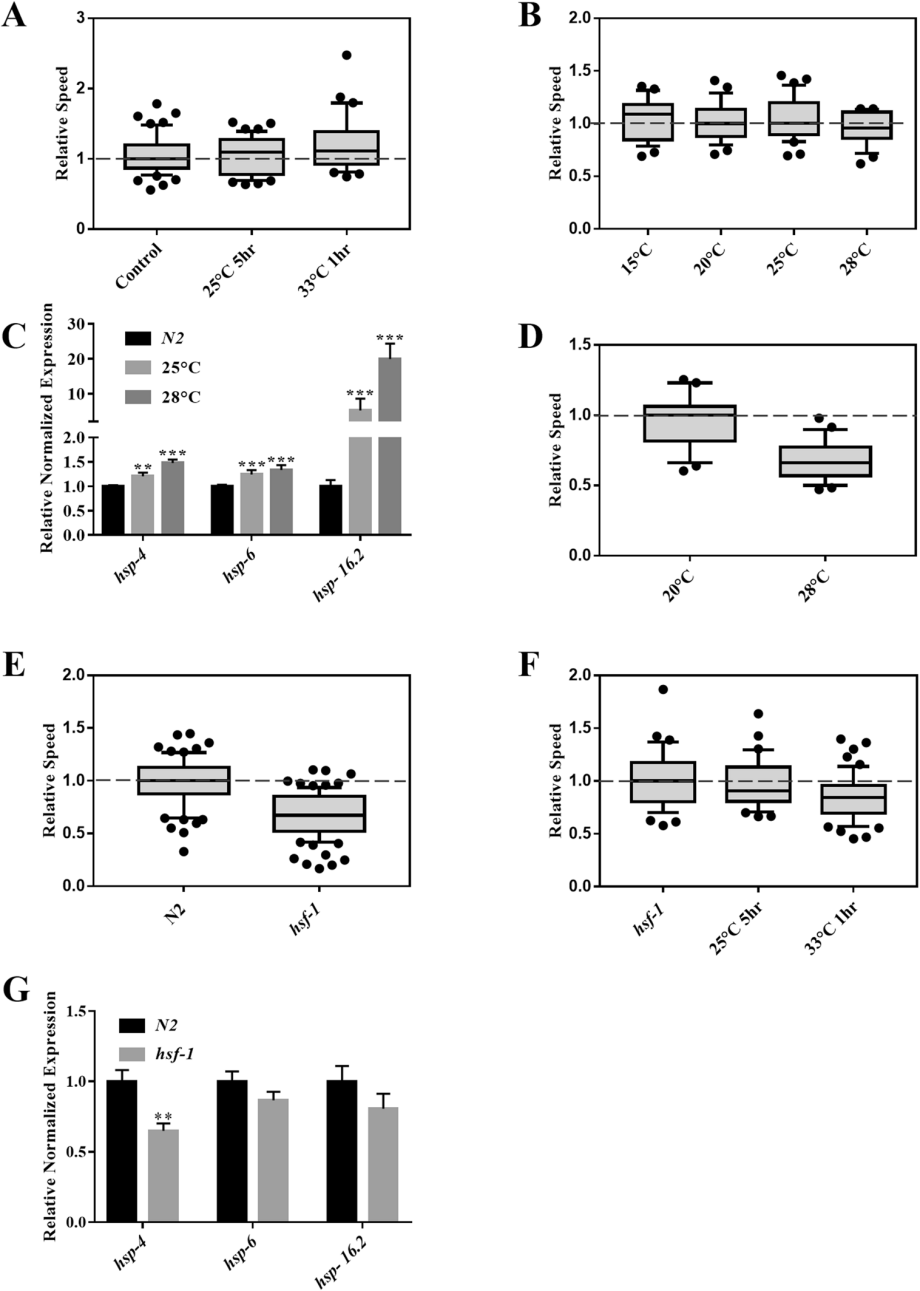
Effect of temperature on electrotactic response. Refer to Fig. 1 for a description of box plot. (**A**) Phenotype of animals exposed to two different heat conditions. No significant difference in speed was observed for any of the treatments (25 °C for 5 h, *p* = 0.970; 33 °C for 1 h, *p* = 0.101). (**B**) Electrotaxis of wild-type day-1 adults following exposure to heat, each for an 8 h period. No significant change was observed in the speed at 15 °C (*p* = 0.9215), 25 °C (*p* = 0.9026) and 28 °C (*p* = 0.3925). (**C**) RT-qPCR analysis of *hsp-4, hsp-6* and *hsp-16.2* in wild-type day-1 adults following 8 h exposures to 25 °C and 28 °C. The expression of the UPR chaperones was significantly increased following both treatments (*p* < 0.001). (**D**) Electrotaxis speed of wild-type animals following a chronic exposure at 28 °C for 3 days. Speed was significantly decreased (*p* < 0.0001) compared to the control grown at 20 °C. (**E**) *hsf-1(sy441)* animals exhibited significant slowness (*p* < 0.001). (**F**) *hsf-1(sy441)* animals exposed to 33 °C for 1 h exhibit significant slowness (*p* = 0.0017) while those exposed to 25 °C for 5 h had no obvious change (*p* = 0.6532)**.** . (**G**) RT-qPCR analysis of *hsp-4, hsp-6* and *hsp-16.2* in *hsf-1* mutants. The expression of *hsp-4* was reduced (*p* < 0.01) however *hsp-6* (*p* = 0.145032) and *hsp-16.2* (*p* = 0.234128) were unaffected. The numbers of animals were (**A**) Control, untreated: n = 57, 25 °C for 5 h: n = 49, 33 °C for 1 h: n = 31. (**B**) 20 °C control: n = 26, 15 °C: n = 29, 25 °C: n = 35, 28 °C: n = 24. (**C**) Control : n = 3 batches; 25 °C: n = 3 batches, and 28 °C: n = 3 batches. (**D**) 20 °C control and 28 °C: n = 23. (**E**) N2: n = 70, *hsf-1(sy441):* n = 90. (**F**) *hsf-1(sy441)* untreated: n = 63, *hsf-1(sy441)* 25 °C for 5 h: n = 51, *hsf-1(sy441)* 33 °C for 1 h: n = 31. (**G**) N2: n = 3 batches; *hsf-1(sy441):* n = 3 batches. Statistical analyses were performed using one-way ANOVA with Dunnett’s post hoc test (A,B,F), Student’s t-test (D,E), and one-way ANOVA with Tukey’s post hoc test (C,G).

Studies have shown that heat treatments can have both positive and negative effects on animals due to activated HSR^7,46^ and UPR^ER^^7,47^. We investigated the electrotactic movement of young adults that were subjected to acute and chronic heat conditions. Worms exposed to two types of heat pulses, namely 5 h at 25 °C and 1 h at 33 °C, showed no change in their speed (Fig. 5A). Three additional treatments with a longer exposure time (i.e., 8 h each at 15 °C, 25 °C, and 28 °C), did not affect the speed either (Fig. 5B); however, all three heat shock chaperons (*hsp-4, hsp-6*, and *hsp-16.2*) were significantly upregulated (Figs. 5C, S4), suggesting the activation of UPR pathways. By contrast, chronic heat treatment at 28 °C for 3 days significantly reduced the speed (Fig. 5D). This demonstrates that unlike shorter duration (up to 8 h) of heat pulses, prolonged heat stress affects the electrotaxis speed of *C. elegans*.

One of the ways by which heat can induce stress is by interfering with the function of the heat shock transcription factor (HSF)^7^. In *C. elegans*, the HSF ortholog *hsf-1* is transcriptionally activated by a variety of stress conditions, including heat exposure^48^. In support of *hsf-1*′s involvement, we found that *hsf-1(sy441)* mutants have a significant electrotaxis speed deficit (Fig. 5E), which was further enhanced by a 1 h heat pulse at 33 °C but not by 5 h at 25 °C (Fig. 5F). There was no change in *hsp-6* and *hsp-16.2* levels in *hsf-1* mutant animals, although *hsp-4* was significantly down-regulated (Fig. 5G). Given that *hsf-1* regulates the expression of a large number of genes^49^, we speculate that other heat shock chaperon(s) might mediate *hsf-1* function in electrotaxis. We also found that the *hsf-1(sy441)* phenotype was exacerbated by PQ-induced stress and L1 starvation (Figs. S5, S6), which indicates that *hsf-1* is a key regulator of diverse stress-induced responses.

### Electrotactic movement decreases with reduced diet but increases after exercise

**Figure 6:**
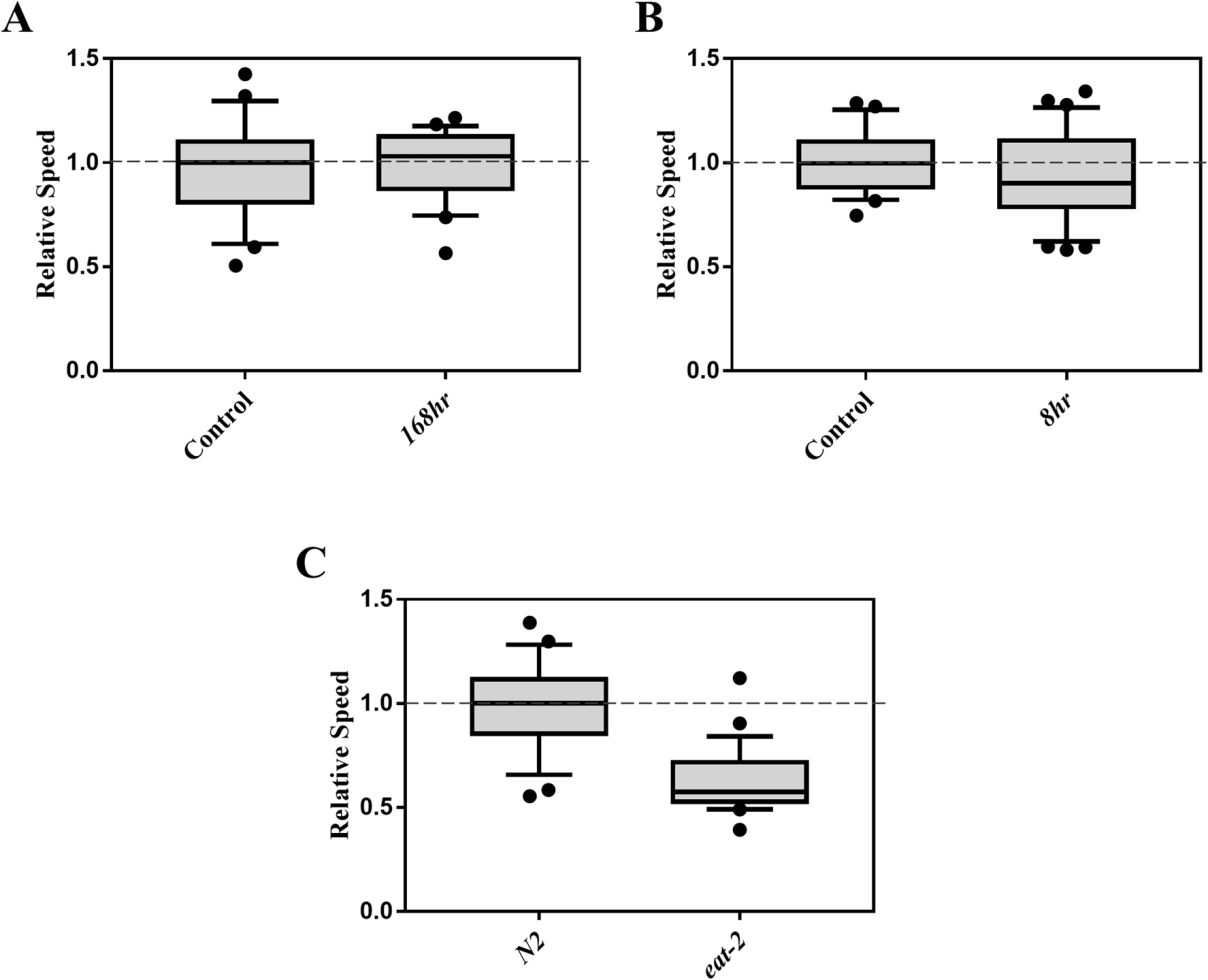
Effect of starvation on the electrotactic movement. Refer to Fig. 1 for a description of box plot. (**A**) Electrotaxis of animals starved for 168 h during the L1 larval stage. The speed was unaffected (*p* = 0.660). (**B**) Electrotaxis of day-1 adult wild-type animals starved for 8 h. The speed was comparable to the control (*p* = 0.3350). (**C**) The speed of *eat-2(ad1116)* adults was significantly reduced (*p* < 0.0001). The numbers of animals were (**A**) Control n = 21, 168 h n = 21. (**B**) Control n = 22, 8 h n = 25. (**C**) N2 day-1 adults n = 25, *eat-2(ad1116)* n = 29. Statistical Analysis was performed using unpaired Student’s t-test.

Another form of stress that we tested is starvation. Initially, the effects of two different starvation exposure protocols on electrotaxis were investigated. In one case, L1 larvae were subjected to starvation for 168 h and subsequently allowed to resume development in the presence of food. In the second case, day-1 adults were exposed to acute starvation conditions. Both nutrient deprivation treatments showed that the effect on electrotaxis was comparable to that of the controls (Fig. 6A,B).

We also investigated a chronic form of nutrient deprivation that involves reduced daily caloric intake. This treatment, termed ‘dietary restriction (DR),’ has been shown to be beneficial in animals such as lifespan extension, increased metabolic fitness, and reduced severity of age-related physiological decline^50–52^. To this end *eat-2* mutants were examined. *eat-2(ad1116)* animals have a longer lifespan that is attributed to chronic DR due to their slower pharyngeal pumping^33^. We found that the mutants had a significantly slower speed, suggesting that DR affects electrotaxis behavior (Fig. 6C). Since *eat-2* mutants show higher fluorescence based on *hsp-4::GFP* reporter analysis^53^ but no change in *hsp-6::GFP*^54^, we conclude that the sensitivity of electrotactic response to dietary restriction is likely due to the detrimental effect of persistent ER stress.

In addition to starvation and DR, we explored the effect of dietary changes on electrotaxis. Two of the bacterial strains that were initially tested were *Streptomyces venezuelae* and *Bacillus thuringiensis*. The metabolites secreted by these bacteria cause intracellular stress in *C. elegans*^55,56^. We found that neither bacteria had an impact on electrotaxis (Fig. S7). Next, worms were fed with two plant pathogens, *Agrobacterium tumefaciens* and *Pectobacterium carotovorum*^57–59^ and a nonpathogenic fungus, *Cryptococcus aquaticus*. Plant pathogens have been reported to have a detrimental effect on the lifespan of worms^60^. None of these cultures had an obvious effect on electrotactic movement (Fig. S8). We also tested three commonly used *E. coli* strains, HB101, HT115, and DH5α, but, once again, observed no change in movement (Fig. S8). These results led us to conclude that electrotaxis behavior is unaffected by the above selected set of microorganisms, although the possibility of reduced nutrient intake in some or all cases cannot be ruled out. In the future, a larger set of microbial diets should be tested to investigate their effect on animals more thoroughly.

**Figure 7:**
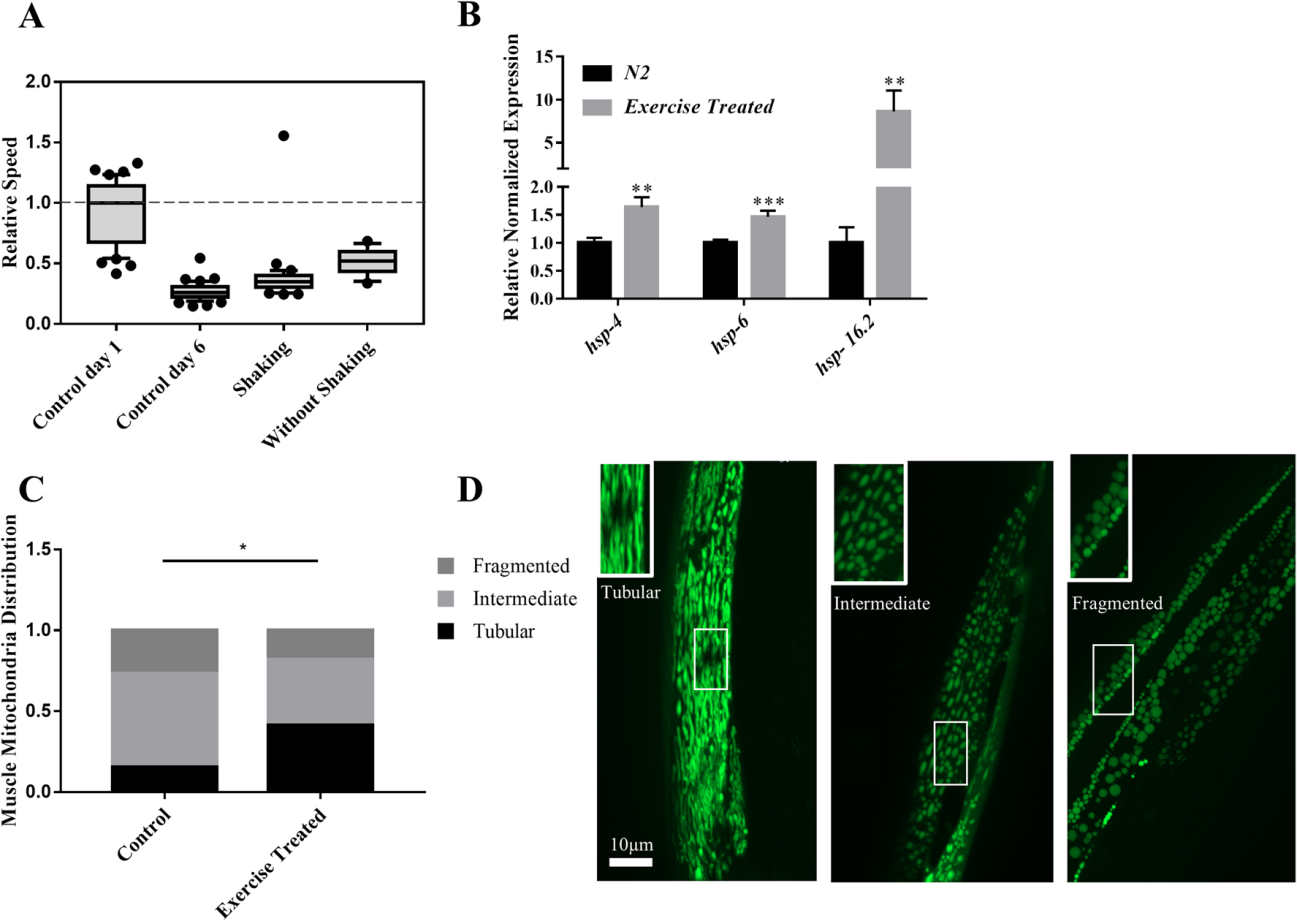
Effect of Exercise on electrotaxis reponse. (**A**) Worms were subjected to swimming exercise. A significant increase in the speed was observed using two different treatments (with shaking and without shaking: *p* < 0.001) compared to same age control. The speed was slower than day-1 controls (*p* < 0.0001). (**B**) RT-qPCR analysis of *hsp-4, hsp-6* and *hsp-16.2* in wild-type adults following 7 days of shaking exercise treatment. The expression of all three chaperones was significantly increased (*p* < 0.01). (**C, D**) Muscle mitochondria morphology in day-6 *myo-3::GFP(mit)* adults after shaking exercise. Animals were placed into three different categories, i.e., having mostly tubular, fragmented, and intermediate (i.e., combination of tubular and fragmented) shapes of mitochondria. These shapes correspond to mitochondrial network being normal (tubular) and defective (fragmented and intermediate). Exercise resulted in significantly more animals having tubular mitochondria (*p* = 0.045). The numbers of animals were: (**A**) Control day-1: n = 41, Control day-6: n = 48, shaking: n = 36 , without shaking: n = 14. (**B**) N2: n = 3 batches, exercise treated: n = 3 batches, (**C**) Control: n = 23, exercise treated: n = 23. Data was analyzed using one-way ANOVA with Dunnett’s post hoc test (A), one-way ANOVA with Tukey’s post-hoc test (B), and Chi-square test (C).

Finally, we investigated the effect of physical activity on the electrotactic movement of animals. The beneficial effects of exercise in humans and model organisms are well documented. In addition to improving muscle fitness, exercise also promotes neuronal health^61–63^. Since swimming activity in *C. elegans* induces known features of exercise reported in mammalian systems^62,64,65^, worms were allowed to swim in M9 for 30 min daily, starting at the L3 larval stage, for a period of 7 days (see Methods for details). The results showed that animals had a significantly faster electrotaxis speed compared to controls of the same age, although they were slower compared to day-1 controls (Fig. 7A). Consistent with this, all three heat shock chaperons, *hsp-4, hsp-6,* and *hsp-16.2*, were upregulated (Fig. 7B). Additionally, we found that exercise caused a significant increase in the proportion of animals with tubular muscle mitochondria^62,66^ (Fig. 7C,D). There was no change in dopaminergic neurons (Fig. S9).

## Discussion

The experiments described herein are the first to demonstrate that the electrotactic response of *C. elegans* is affected by mutations and environmental conditions that increase cytosolic, mitochondrial, and ER stress. We found that the electrotaxis speed was altered by treatment with PQ and tunicamycin, two chemicals that are known to negatively impact the locomotory behavior of worms on solid media^17,67^. PQ exposure causes generation of ROS, which activates the expression of multiple chaperones such as *hsp-4* (ER stress), *hsp-6* and *hsp-60* (both mitochondrial stress), and *hsp-16.2* (cytosolic stress). The effects of PQ on mitochondrial and ER stress are well documented; hence, the effects of PQ on electrotaxis are likely due to increased stress associated with these two organelles^13,30,68^.

Independent of ROS, our data show that mutations that cause mitochondrial stress also lead to decreased electrotaxis speed. The slow speed of *atfs-1(gf)* mutants indicates that the UPR^MT^ contributes to maintaining the wild-type behavior. Analysis of *ucp-4, mev-1,* and *pink-1* mutants revealed that exposure to a relatively low concentration of PQ induced speed defects. In mammalian cells, mutations in UCP genes are associated with increased ROS and oxidative stress^69–71^. Interestingly, Iser and colleagues (2005) reported earlier that *ucp-4(ok195)* worms exhibit wild-type lifespan and survival in the presence of PQ, although the animals display an elevated ATP level^36,72^. Our finding that *ucp-4* mutants have defects in electrotaxis suggests that the movement response of animals may be sensitive to ROS levels. Future studies directly measuring oxidative damage following PQ exposure in different genetic backgrounds will be helpful in illuminating the relationship between ROS and electrotaxis.

*isp-1* and *gas-1 mutants* exhibit reduced electrotaxis speed. While mutations in both these genes are also associated with electron transport hindrance and increased ROS generation, the *isp-1* mutant actually exhibits oxidative stress resistance due to low oxygen consumption and increased expression of mitochondrial superoxide dismutase SOD-3^36^. On the other hand, *gas-1* mutants are hypersensitive to oxidative stresses such as PQ^36,37^. The lifespan phenotypes of *isp-1* and *gas-1* mutants are also opposite, wherein *isp-1* animals have a long lifespan and *gas-1* are short-lived, possibly due to differences in ROS production and ROS-associated enzymatic activity^36^. These data suggest that electrotaxis speed is independent of pathways involved in lifespan maintenance. The *isp-1(qm150)* and *gas-1(fc21)* also both activate the UPR^MT^^73,74^, which led us to investigate the role of *atfs-1*. We found that *atfs-1* null mutant and gain-of-function mutant had a slower speed in our assay. Moreover, RNAi knock-down of *atfs-1* reduced the speed of *isp-1* mutant but not *gas-1* mutant animals. Together with known roles of *atfs-1* in regulating the expression of mitochondrial and cytosolic heat shock chaperons, these results support the conclusion that multiple stress response signaling affect the electrotaxis behavior of *C. elegans*.

The effect of ROS induced by PQ and mitochondrial mutants could also impact UPR^ER^. Thus, we investigated the responses of animals treated with tunicamycin and observed defects in electrotaxis speed that were accompanied by an increase in the expression of the ER chaperone GRP78/BiP homolog *hsp-4*. Similar electrotaxis phenotypes were also observed in UPR^ER^ mutants, indicating that the ER genes play a crucial role in mediating normal movement behavior. The analysis of mutants affecting the three major arms of UPR^ER^ revealed that while *ire-1, pek-1,* and *xbp-1* are needed to maintain a normal response, *atf-6* is not essential. Differences between UPR^ER^ pathway components in mediating some of the biological processes have been reported earlier. Shen et al. (2001) found that while knockdown of *pek-1* and *ire-1* affected larval development, there was no such phenotype in the case of *atf-6*^75^. Additionally, *ire-1* and *xbp-1* mutants have a short lifespan and slower development, but *pek-1* and *atf-6* mutants appear normal^56,76^. Finally, animals lacking *ire-1, xbp-1*, and *atf-6* function show high sensitivity to pore forming bacterial toxin Cry5B but *pek-1* does not appear to be involved^46^. In spite of these differences, we found that increasing the ER stress load by tunicamycin treatment exacerbated the electrotaxis defects of all three UPR^ER^ pathway mutants.

In addition to the known UPR^ER^ genes, we also tested the less well-characterized gene *pqe-1*. Mutations in *pqe-1* are thought to cause proteotoxic ER stress, possibly due to the upregulation of global protein synthesis^77^. Consistent with this idea, our results showed that *pqe-1* mutants have an abnormal electrotactic response. Studies in yeast have shown that the REXO1 (RNA exonuclease homolog 1) domain of PQE-1 functions in the processing of the 3′ ends of rRNA and tRNA^78,79^, which may suggest a role of *pqe-1* in the regulation of protein translation. Since *pqe-1* localizes to the cell nucleus^23,24^, it is possible that it affects genes whose products modulate the function of translation machinery. To investigate this further, we knocked-down the eIF4G homolog, *ifg-1*, a component of the initiation factor 4F (eIF4F) complex^45^, but saw no suppression of the *pqe-1* mutant phenotype. This result combined with high sensitivity of mutant animals to tunicamycin and changes in the expression of multiple chaperons and *xbp-1* lead us to suggest that *pqe-1* plays an essential role in maintaining both HSR and organelle-specific stress response (UPR) pathways in *C. elegans*.

One of the ways by which stress can affect electrotaxis is by causing damage to neurons. Consistent with this, animals exposed to PQ and tunicamycin exhibited degenerated dopaminergic neurons (Fig. S1). A similar phenotype was also observed in *pqe-1* mutants (Fig. S2). These findings, along with our previous work demonstrating the role of dopaminergic neuron signaling in mediating electrotaxis behavior^22^, led us to conclude that neuronal defects may in part contribute to the electrotaxis phenotype in animals as a result of stress-inducing conditions.

In addition to examining the effects of genetic mutations and compounds, we investigated whether environmental perturbations alter the electrotactic response. Among other effects , heat is reported to induce mitochondrial stress^7^. High temperatures have also been shown to modulate ER and cytosolic stress. This may involve multiple mechanisms, such as activation or repression of PERK depending on the severity of the heat regimen^47^ and changes in global protein synthesis and apoptosis^47^. We exposed animals to acute heat and found that their electrotaxis was unaffected. Chronic heat, however, severely reduced the speed, suggesting that behavior is affected by prolonged heat exposure, which is consistent with cellular stress playing a role in modulating electrotaxis. The resistance to heat-induced stress appears to depend on the transcription factor *hsf-1*, since *hsf-1* mutants exhibit electrotaxis defects and are sensitive to heat treatments.

As diet can also play a role in the UPR maintenance^53,80^, we subjected worms to different forms of starvation. When *C. elegans* are starved during the L1 larval stage, they enter L1 arrest, which halts the development and reproductive growth, enhances stress resistance, modifies feeding behavior, and alters metabolic flux^81,82^. Starvation during the L4 larval and adult stages has beneficial effects such as extended life span, germline cell reduction, delayed reproduction, and thermotolerance^83^. At the cellular level, starvation induces both mitochondrial and ER stress^53,80,84^. Mitochondria become fragmented in starved animals and are cleared through mitophagy^10,80,85^. Zhang et al.^86^ reported that starvation caused ER stress and led to activation of PERK/eIF2α in an astrocyte cell line. Other groups have shown changes in the regulation of different ER proteins, such as chaperones^84^. Our work demonstrates that short-term DR does not affect the electrotactic response. However, *eat-2* mutants that are chronically starved, and therefore mimic a long-term DR condition, show a significantly slower electrotaxis speed. It is conceivable that a reduction in speed may aid in the long life span of *eat-2* mutants as the animals are slow feeders^53^. This line of reasoning agrees with previous findings that young adults lacking *eat-2* function move slower on solid media^33^. Furthermore, *eat-2* mutants show increased expression of *hsp-4*^53^ but not *hsp-6*^54^, suggesting higher ER stress levels.

The last stress condition we investigated was a daily exercise regimen. The benefits of exercise on muscular and neuronal health are well demonstrated. Healthier muscles through exercise allow animals to increase their athletic capacity^63^. At the molecular level, exercise activates the UPR^ER^ response as a protective mechanism^87^. Beneficial effects of exercise have also been demonstrated in *C. elegans*, such as a longer lifespan and improved neuronal and muscular health^62,64,65,88^. Laranjeiro et al. (2017) reported that the ER chaperone *hsp-4* is downregulated in animals subjected to exercise, which suggests a conserved role of UPR^ER^ in exercise-induced benefits^64^. Our lab showed earlier that defects in muscles reduce the electrotaxis speed of *C. elegans*^22^. Consistent with the above studies, we found that daily exercise treatment significantly improved the electrotaxis speed of *C. elegans* and resulted in increased expression of heat shock chaperons as well as better preservation of mitochondrial morphology.

In summary, the work described in this paper shows that electrotactic movement of worms is reduced by mutations in genes that affect HSR, UPR^MT^, and UPR^ER^ as well as following chronic exposure to chemicals, heat, and reduced diet. In contrast, short bursts of daily exercise are beneficial since they resulted in a higher speed of animals and better preservation of mitochondrial morphology. Collectively, these findings lead us to conclude that conditions that elevate cellular stress for prolonged periods cause detrimental effects, whereas transient stress imparts a beneficial effect on health. The results established that a microfluidic-based assay is a reliable output of locomotory circuits and demonstrate that electrotaxis can be used to study stress response pathways in *C. elegans*.

## Materials and methods

### Strains and culturing

This study used the following *C. elegans* strains: N2: wild-type Bristol isolate; MQ887: *isp-1(qm150)*; CW152: *gas-1(fc21)*; TK22: *mev-1(kn1)*; CY121: *ucp-4(ok195)*; DY356: *pink-1(ok3538)*; GA184: *sod-2(gk257)*; GA186: *sod-3(tm760)*; GA480: *sod-2(gk257); sod-3(tm760)*; QC115: *atfs-1(et15)*; VC3201: *atfs-1(gk3094)*; RB545: *pek-1(ok275)*; RB772: *atf-6(ok551)*; RE666: *ire-1(v33)*; SJ17 *xbp-1(zc12) III; zcIs4 V*; SJ4100: *zcIs13(hsp-6::GFP)*; SJ4058: *zcIs9(hsp-60::GFP)*; PS3551: *hsf-1(sy441*); DY693: *pqe-1(ok1983);zcIs4 V;* RB1611: *pqe-1(ok1983)*; SJ4005: *zcIs4(hsp-4::GFP)*; DY542: *pqe-1(ok1983); bhEx138[pGLC72(Cel-dat-1p::YFP)];* CL2070: *dvIs70[hsp-16.2::GFP* + *rol-6(su1006)]*; DY353: *bhEx138[pGLC72(Cel-dat-1p::YFP)];* SJ4103: *zcIs14[myo-3::GFP(mit)]* and DY356 (5x outcrossed RB2547): *pink-1(ok3538)*. RB2547 and all other strains were originally obtained from the Caenorhabditis Genetics Center (University of Minnesota, St. Paul, MN)^89^.

Except where indicated, animals were grown and maintained at 20 °C on nematode growth medium (NGM) agar plates containing *E. coli* OP50 culture using previously described methods^15^. All experiments used age-synchronous populations obtained by bleach treatment^15^. Experiments involving wild-type and untreated controls were done using young adults unless stated otherwise. Growth times for all animals bearing mutations or undergoing treatments that affect developmental rate were appropriately adjusted.

### Assays and treatments

The following methods were used to analyze worms in this study. Additional details are provided in supplementary methods.

#### Chemical treatments

Paraquat dichloride was obtained from Sigma-Aldrich (St. Louis, MO, USA) and tunicamycin was obtained from Bioshop Canada Inc (Burlington, ON, CA). Paraquat treatments were first prepared as 20 × solutions in M9 buffer; subsequently, 1 × treatment plates were produced by spreading 500 μL of the 20 × solutions across the surface of plates containing 10 mL of NGM agar. Tunicamycin treatments were first prepared as 5 mg/mL stock solutions in 100% DMSO, then diluted with water to make 20 × solutions; subsequently, 1 × treatment plates were produced by spreading 500 μL of the 20 × solutions across the surface of plates containing 10 mL of NGM agar. For chemical treatments, synchronized worms were grown on chemical-containing plates from L1 until adulthood. Young day-1 adults, based on direct observation, were used for experiments.

#### Heat stress

Animals were first grown at 20 °C until young adulthood. Plates were then sealed with parafilm and exposed to specific temperatures in a water bath for desired durations. Control plates were also sealed but kept at 20 °C. After the treatment, parafilm was removed and animals were allowed to recover at 20 °C for 30 min to an hour before electrotaxis assays. For 28 °C treatment, synchronized larvae were allowed to grow at 20 °C till day-1 adulthood and then shifted to 28 °C for 3 days.

#### Feeding bacterial and fungal cultures

*C. elegans* plates were seeded with overnight grown cultures of microorganisms. For *E. coli*, plant pathogens and fungus, L1 animals were transferred on plates and allowed to grow till adulthood. Day-1 adults were used for electrotaxis assays. In the case of *Streptomyces venezuelae* and *Bacillius thuringiensis,* L1 larvae were first fed with *E. coli* OP50 till adulthood. One day-old adults were transferred on maltose yeast extract medium (MYM) plates containing culture of *S. venezuelae* for 24 h. *Bacillus thuringiensi*s culture was grown on NGM agar and fed to one day-old adults for 24 h. Electrotaxis was performed the following day. Control worms were grown in the presence of OP50 culture on NGM-Agar and MYM-Agar plates.

#### Starvation

Animals were first grown on OP50 until day-1 adulthood. They were then transferred to an unseeded NGM agar plate (no food) for 8 h to mimic acute starvation. Following the starvation period, electrotaxis assay was performed.

#### Exercise

Worms were subjected to exercise starting the L3 larval stage until day-6 adulthood (for a total of 7 days). Two different treatments were performed, each of which consisted of 30 min of daily swimming, followed by recovery on LB-agar plates. The first exercise regimen involved a belly dancer shaker in which worms, suspended in M9, were subjected to 100 rpm rotation. The second regimen consisted of keeping worms in M9 on the countertop without shaking, thereby allowing them to perform natural swimming.

### Microchannel fabrication and electrotaxis assay

Microfluidic channels were fabricated as previously described^21,90^. The channel design was printed on a transparency sheet using high-resolution photoplotting to create a photomask, which was then used in conjunction with SU-8 100 negative photoresist (MicroChem Corp., MA, USA) to lithographically pattern the design onto a silicon wafer. Microchannels were then casted by pouring polydimethylsiloxane (PDMS) pre-polymer (Sylgard 184 Kit, Dow Corning Corp., MI, USA; 10:1 ratio of base and cross-linker) onto the resultant master mold and allowing 24 h for curing. The channel was then excised from the PDMS replica and fluid access ports were punched into each end. Next, the channel, a blank PDMS strip and a glass slide were oxidized via exposure to oxygen plasma for 40 s at 40 W power and stuck together to seal the microchannel. Lastly, plastic tubing and insulated copper wire were affixed to the punched reservoirs and secured with PDMS pre-polymer.

The electrotaxis assay protocol has also been described^21,90^. A syringe was attached to one of the inlet/outlet tubes of the PDMS microchannel to facilitate worm loading at the other tube. A power supply was connected via insulated copper wiring to the electrodes of the microchannel device to provide worms with electrical stimulus. A microscope, camera and monitor allowed visualization and recording of the electrotaxis experiment.

In preparation for the assay, worms were washed off of their culture plates, cleaned, and suspended in M9 buffer. Animals were then aspirated into the channel using the syringe pump. Both tubes were then laid flat at the same elevation to eliminate pressure-induced flow. Next, a 3 V/cm DC electric field was applied, and the worm’s resultant behaviour recorded by camera.

Electrotaxis was carried out for up to 5 min. Animals were allowed to travel a minimum distance of 5 mm in one direction, towards the cathode, after which the field polarity was reversed to induce a turning response. Locomotory data was extracted from recorded videos using custom MATLAB-based worm tracking software. Electrotaxis speed data was plotted in box plots.

### Fluorescence microscopy and quantification

Animals were mounted on 2% agar pad containing glass slides. Before placing the cover slip, they were anesthetized using a 15 μL drop of 30 mM NaN_3_ mixed with a drop of M9. GFP fluorescence was visualized using a Zeiss Observer Z1 microscope equipped with an Apotome 2 and X-Cite R 120LED fluorescence illuminator. Fluorescence intensity was quantified using NIH ImageJ (http://rsbweb.nih.gov/ij/). Neurodegeneration was manually scored by counting the number of cell bodies of DAergic neurons and by assessing neurite morphologies and trajectories. Worms consist of two pairs of cephalic neurons (CEPs), a pair of anterior deirid neurons (ADE) and one pair of posterior dieirid neurons (PDE).

### RNAi

The *atfs-1* RNAi plasmid (pGLC171) was constructed by inserting a 759 bp genomic fragment into the L4440 vector using the restriction enzymes *Kpn*I and *Xba*I. The fragment was obtained by PCR using the forward primer GL1649 (5'-AAGGGTACCCACTACTTGGAGAGCGACGAC-3') and reverse primer GL1650 (5'-AAGTCTAGACTACTTCTTGGAACTCCCTGC-3'). We used the RNAi gene silencing protocol described by Wu et al.^41^ Since authors reported that knock-down in parental *isp-1* mutant worms caused an arrest of F1 larvae, L4-stage animals were treated with RNAi and day-2 old adults were analyzed. Synchronized worms were grown on OP50 until L4 stage and then transferred to RNAi plates containing either L4440 empty plasmid or *atfs-1* RNAi plasmid. *atfs-1* knock-down was confirmed by qPCR, which showed a range of ~ 2 × to 10 × reduction in different batches. For the *ifg-1* RNAi, animals were fed with bacteria from Ahringer library. In this RNAi paradigm synchronized animals were grown on OP50 until day-1 adult stage and then transferred to *ifg-1* RNAi plates for 48 h and analyzed right after.

### Quantitative reverse transcription PCR (RT-qPCR)

For RT-qPCR experiments, synchronized cultures were prepared by bleaching adult hermaphrodites twice in two successive generations to obtain highly synchronous cultures. After the second round of bleaching, eggs were grown until the day-1 adulthood. Total RNA was extracted from these animals using trizol (Catalog Number T9424, Sigma-Aldrich, Canada), according to the manufacturer’s instructions. The SensiFAST cDNA Synthesis Kit (Catalog Number BIO-65053, MeridianBioscience, Canada) was used to obtain cDNA according to manufacturer’s instructions. RT-qPCR was performed (in triplicate) using the Bio-Rad cycler CFX 96 machine. The primers are listed in the Supplementary table (Table S1). The reaction was set up using SensiFAST SYBR Green Kit (Catalog Number BIO-98005, BIOLINE, USA) according to the manufacturer’s instructions. The expression levels of the analyzed genes were normalized to *pmp-3*.

### Data analysis

GraphPad Prism 7 was used to plot the graphs. For all assays, data from repeat experiments were pooled and analyzed together. Statistical analyses were done using GraphPad Prism 7 except for RT-qPCR experiments that were analyzed using CFX Maestro 3.1 software (Bio-Rad, Canada; https://www.bio-rad.com/en-ca/product/cfx-maestro-software-for-cfx-real-time-pcr-instruments). P values less than 0.05 were considered statistically significant.

## Supplementary Information

Supplementary Information

## Data Availability

All data generated or analysed during this study are included in this published article (and its Supplementary Information files). Additional details are available from the corresponding author on reasonable request.
