# Supplementary material for: C. elegans electrotaxis behavior is modulated by heat shock response and unfolded protein response signaling pathways
Source: Sci Rep. 2021 Feb 4;11:3115. doi: 10.1038/s41598-021-82466-z (PMC7862228; doi:10.1038/s41598-021-82466-z)
Supplement: Supplementary file 1 — Supplementary Information [file 41598_2021_82466_MOESM1_ESM.docx]

**Supplementary Information**

***C. elegans* electrotaxis behavior is modulated by heat shock response and unfolded protein response signaling pathways**

Shane K. B. Taylor^1$^, Muhammad H. Minhas^1$^, Justin Tong^1^, P. Ravi Selvaganapathy^2^, Ram K. Mishra^3^, and Bhagwati P. Gupta^1*^

^1^Department of Biology, McMaster University, Hamilton, ON

^2^Department of Mechanical Engineering, McMaster University, Hamilton, ON

^1^Department of Psychiatry and Behavioural Neurosciences, McMaster University, Hamilton, ON

**Supplementary Methods**

**Lifespan Assay**

Lifespan analysis was carried out at 20^0^C on NGM agar plates with *E. coli* OP50 bacteria. Each strain was repeated at least twice. Each batch contained a minimum of 30 animals per strain and worms were scored for viability every day, from day 1 of adulthood. Young adult worms were transferred to fresh plates every other day and the numbers of dead worms were recorded.

GraphPad Prism software was used to calculate p values using the log-rank (Kaplan-Meier) method.

**Dopaminergic neuron analysis**

Neuronal phenotype was visually scored by counting cell bodies of dopaminergic (DA) neurons and observing dendritic morphologies under a Nomarski fluorescence microscope. In wild-type animals, three pairs of DA neurons (two pairs of CEPs and one pair of ADE) and their smooth dendritic/axonal projections are visible in the head region. Mutations and drug-treatments that cause defects in neurons result in fewer cell bodies and/or projections showing abnormal phenotypes such as blebbing, punctate pattern, deformed shape, faint appearance. and complete absence. Such animals were considered defective. Multiple batches of animals were scored on different days and from different culture plates to ensure that results were unbiased.

**Supplementary Tables**

**Supplementary Table S1.** List of primers used in this study.

| **Primer** | **Gene** | **Direction (forward, FP; reverse, RP)** | **Sequence** |
| --- | --- | --- | --- |
| GL747 | *pmp-3* | FP | CTTAGAGTCAAGGGTCGCAGTGGAG |
| GL748 | *pmp-3* | RP | ACTGTATCGGCACCAAGGAAACTGG |
| GL1446 | *xbp-1* | FP | CGCAGCCCAAAATGCTAGAG |
| GL1447 | *xbp-1* | RP | AGATCGCGCATCACATCCTC |
| GL1633 | *atfs-1* | FP | CGAGCCGAGAAGAAGGGAAG |
| GL1634 | *atfs-1* | RP | GCGCCCATTTTACGAAGCTC |
| GL1643 | *hsp-4* | FP | AAGCTTCTGAGGAGCCATCG |
| GL1644 | *hsp-4* | RP | GGGGTTGGGTTGGGAAAGAA |
| GL1645 | *hsp-6* | FP | AACCATTGAGCCATGCCGTA |
| GL1646 | *hsp-6* | RP | CTTGAACAGTGGCTTGCACC |
| GL1647 | *hsp-16.2* | FP | GTCCAGCTCAACGTTCCGT |
| GL1648 | *hsp-16.2* | RP | TCTCAGAAGACTCAGATGGAGAGAT |
| GL1685 | *ifg-1* | FP | CAGCAGCCATACCGTGGAAG |
| GL1686 | *ifg-1* | RP | TCGAAGGTCTAACAACTGGTGG |

**Supplementary Figures**

**Supplementary Figure S1.**

Dopaminergic (DA) neurons in young adult *dat-1::*YFP transgenic animals treated with PQ and Tunicamycin. (**A)** Quantification of neuronal degeneration following exposure to tunicamycin at different concentrations. Tunicamycin exposure caused a significantly higher dopaminergic neurodegeneration compared to untreated controls (2µg/mL p = 0.0185, 5µg/mL p = 0.0058, 10µg/mL p = 0.0008). (**B)** Quantification of neuronal degeneration following exposure to PQ at different concentrations. 125 μM PQ had no significant degeneration (p = 0.9996) but significant degeneration can be seen at 250µM (p = 0.0079). (**C-E)** Neuronal morphologies as revealed by *dat-1p::yfp* expression Arrows point to cell bodies and dashed lines represent the boundary of the head. The animal in C has normal number of neurons (3 pairs) and smooth appearance of dendritic/ axonal projections. Panels D and E show animals that are defective. In one case (D) only five cell bodies are visible. The dotted regions represent few example areas of neurodegeneration where projections can be seen having blebbing and punctate appearances. Untreated control animals **(C)**, 250 μM PQ **(D),** and 10 μg/mL tunicamycin **(E)**. Exposure to tunicamycin caused damage to dopaminergic neurons as seen by punctate and missing axons. The numbers of animals examined were: **(A)** 25 for *dat-1::YFP* untreated, 53 for Tuni 2 μg/mL, 47 for Tuni 5 μg/mL, and 42 for Tuni 10 μg/mL. (**B)** 25 for *dat-1::YFP* untreated, 27 for PQ 125 μM, and 30 for PQ 250 μM. Data in A and B was analyzed using one-way ANOVA followed by Dunnett’s post hoc test.

**
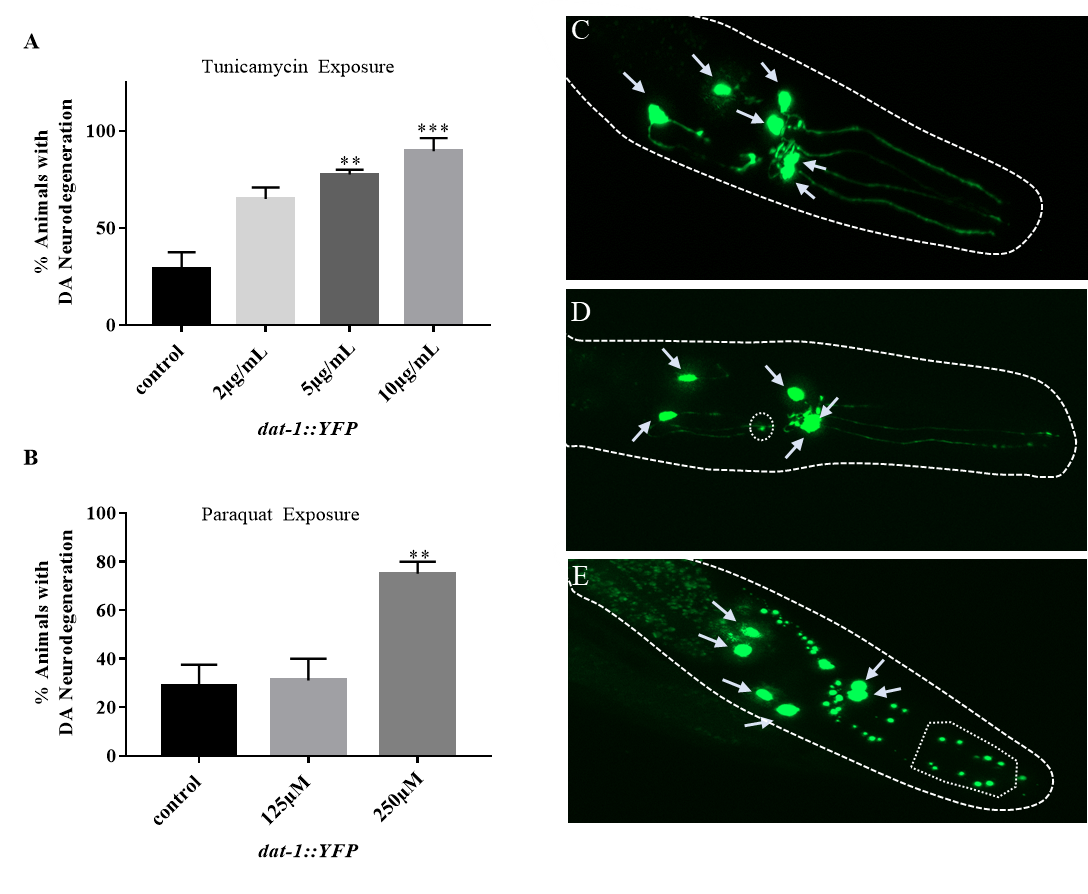
**

**Supplementary Figure S2. (A)** *hsp-4::GFP* reporter analysis in *pqe-1(ok1983)* animals showed an increase in GFP fluorescence (p=0.0086, Student’s t-test). **(B)** Quantification of neuronal degeneration in *pqe-1* mutants on day 1 and 3 of adulthood using the *dat-1::*YFP marker**.** Neuronal defects in mutant animals increase with age. (**C)** *pqe-1**(ok1983)* animals have a shorter lifespan (mean 15.2± 0.6) compared to *N2* (mean 15.9 ± 0.6) (p = 0.0222). The numbers of animals examined were: **(A)** *N2*: n = 24, *pqe-1(ok1983)* n = 18. **(B)** *Control day 1*: n = 47, *pqe-1(ok1983)* – day 1: n = 45, Control day 3: n = 50, *pqe-1(ok1983)* – day 3: n = 51. **(C)** *N2*: n = 66, *pqe-1(ok1983)* n = 80.


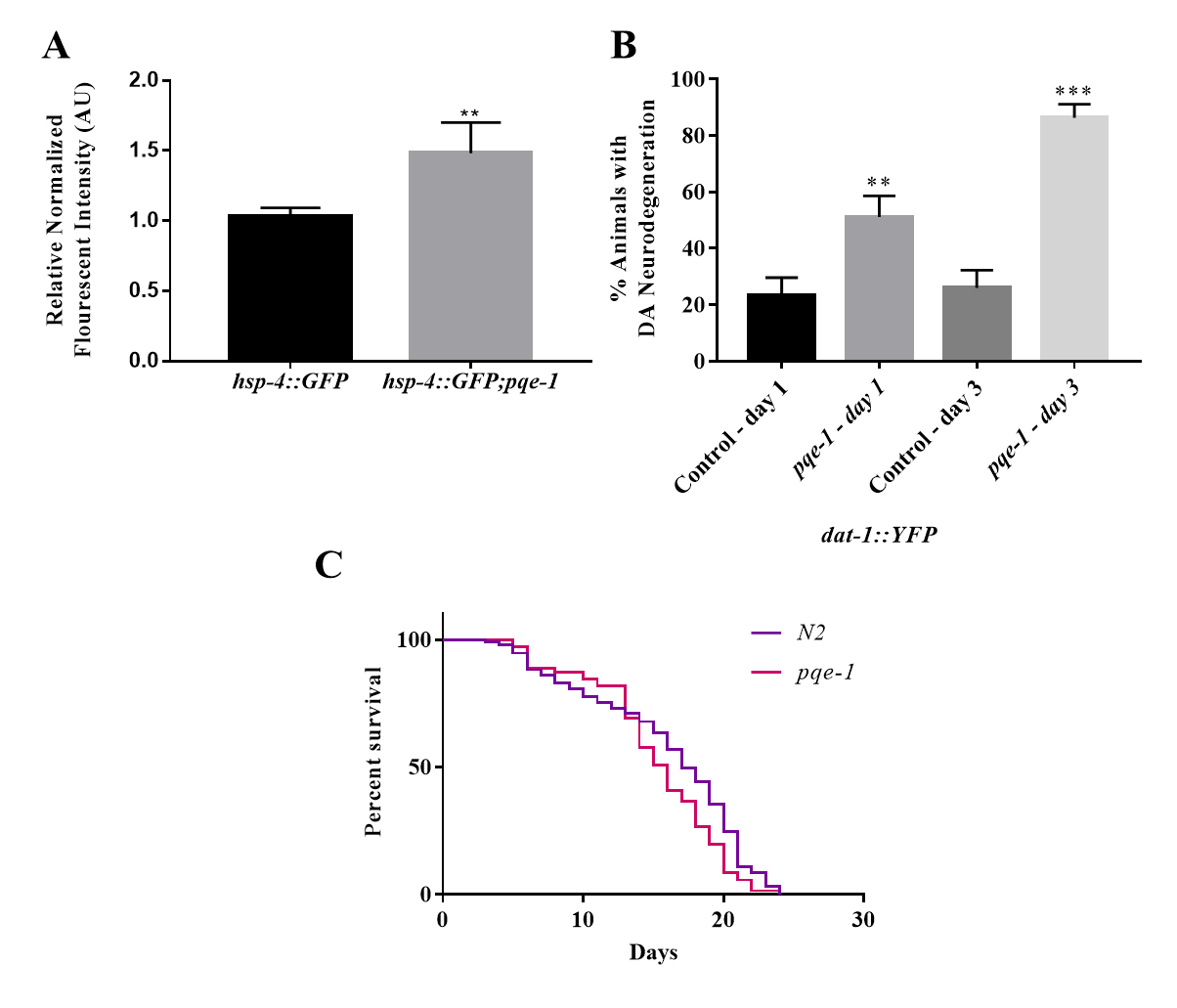


**Supplementary Figure S3.** RT-qPCR analysis the UPR chaperones following *ifg-1 RNAi*. The knock-down was confirmed by measuring *ifg-1* levels. **(A)** In N2 animals, *hsp-4* was unchanged. *hsp-6* was downregulated but *hsp-16.2* was increased roughly three folds (p = 0.002 and 0.03, respectively). **(B)** *ifg-1* RNAi in *pqe-1(ok1983)* animals had no effect on *hsp-4* but both *hsp-6* and *hsp-16.2* were upregulated (p = <0.001 and 0.013, respectively). While the increase in *hsp-16.2* is lower than *pqe-1* mutant alone (see Figure 4A), it is nevertheless up by 5-6 folds. Samples included: L4440 control: n = 2 batches, *ifg-1* RNAi: n = 2 batches, *pqe-1(ok1983),* L4440 control: n = 2 batches, *pqe-1(ok1983);* *ifg-1* RNAi: n = 2 batches. Data was analyzed using one-way ANOVA with Tukey’s post hoc test.


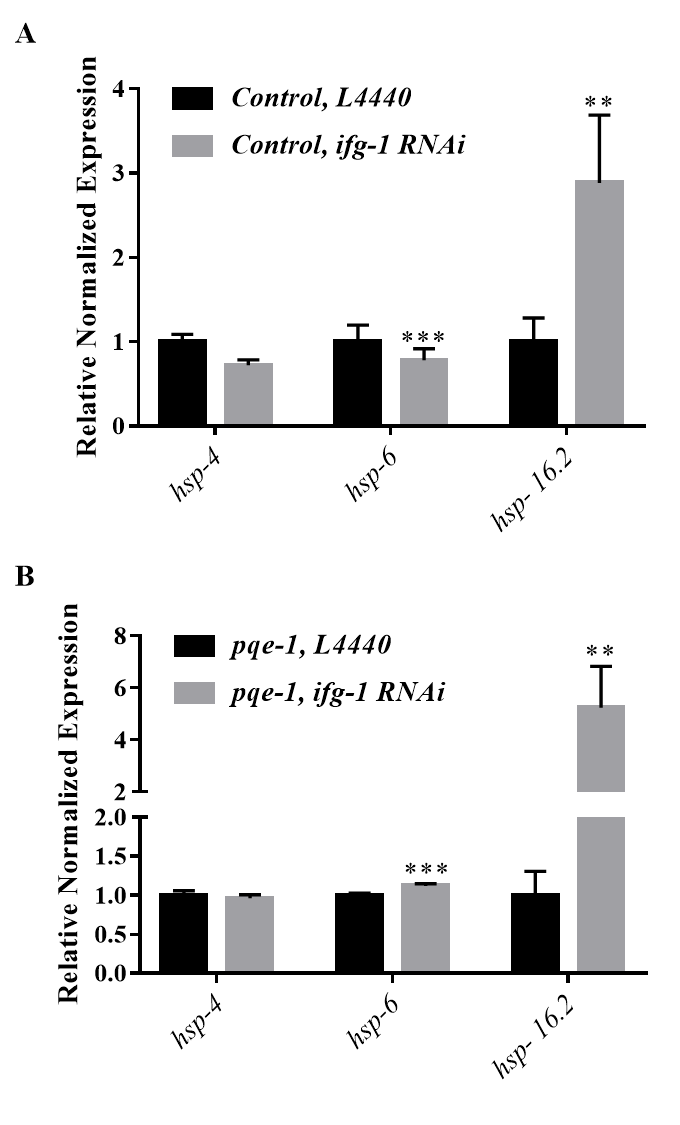


**Supplementary Figure S4.** Analysis of *hsp-4::GFP* reporter in wild-type N2 day-1 adults following 8 hrs exposures to 25°C and 28°C . The fluorescence was significantly increased for both temperatures (p=0.0001). Data was analyzed using one-way ANOVA followed by Dunnet’s post hoc test. Control *hsp-4::GFP* : n = 20; 25°C: n = 22, *and* 28°C: n = 21.


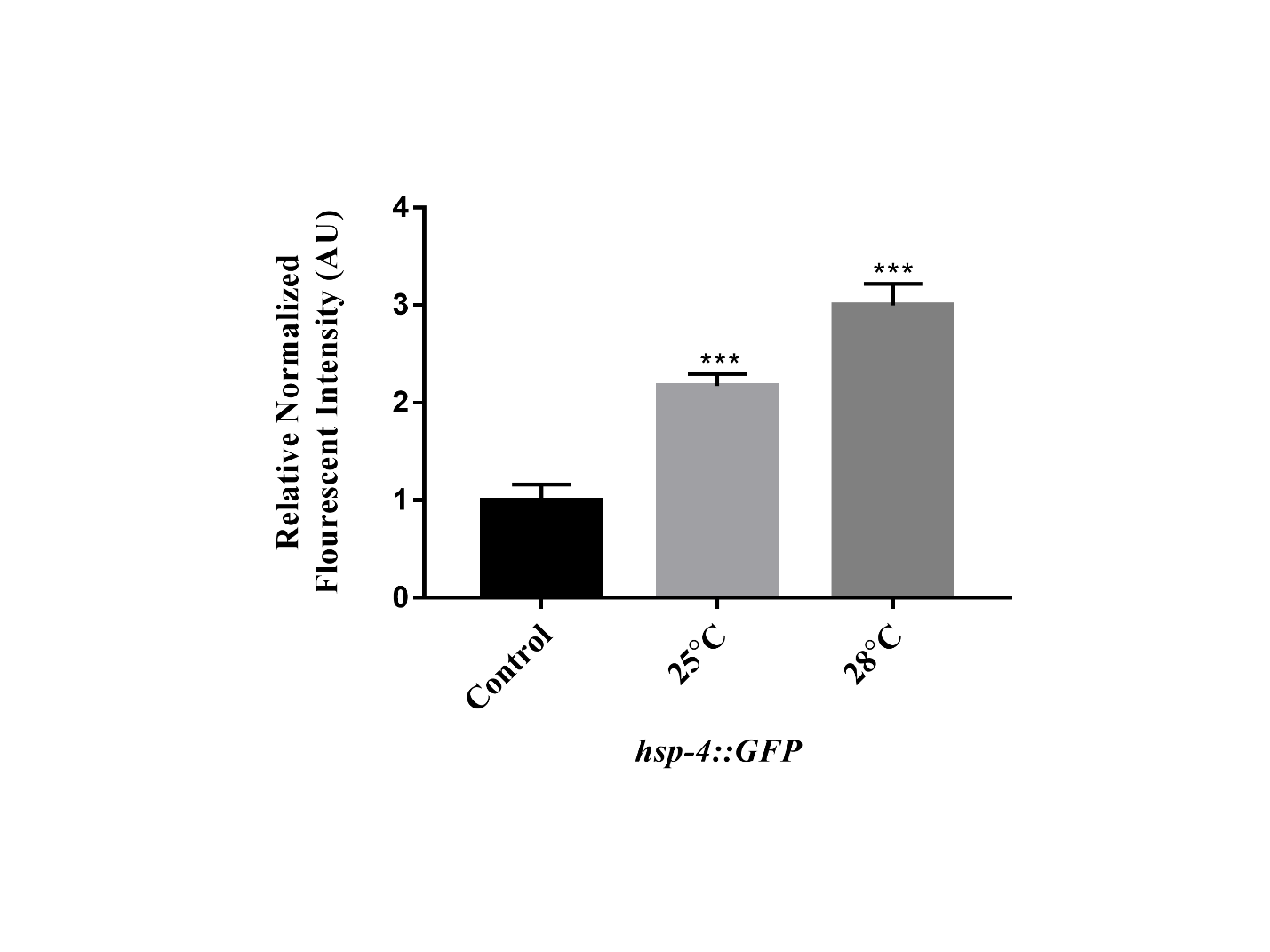


**Supplementary Figure S5.** Electrotaxis of PQ-treated *hsf-1* mutants. Refer to Figure 1 for a description of box plot. Speed of *hsf-1(sy441)* animals is not affected by treatment with 75 μM PQ (p = 0.7901) but is further depressed by treatment with 125 μM PQ (p < 0.001). Data was analyzed using one-way ANOVA followed by Dunnett’s post hoc test. The numbers of animals examined were: *hsf-1(sy441)* untreated: n = 70, *hsf-1(sy441)* + 75 μM PQ: n = 20, *hsf-1(sy441)* + 125 μM PQ: n = 46.


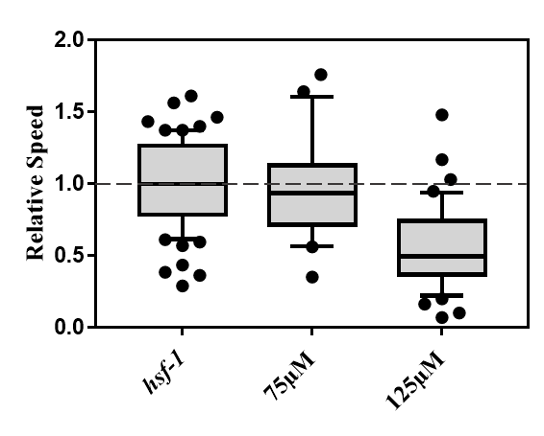


**Supplementary Figure S6.** Electrotaxis of adult *hsf-1* animals that have been starved in M9 for variable lengths of time at L1. Refer to Figure 1 for a description of box plot. *hsf-1(sy441)* animals arrested at L1 for 48 h or 72 h show significant slowness relative to control animals that were starved at L1 for 24 h (p < 0.001 in both cases). The numbers of animals examined were: *hsf-1(sy441)* starved 24h: n = 37, *hsf-1(sy441)* starved 48h: n = 24, *hsf-1(sy441)* starved 72h: n = 27. Data was analyzed using one-way ANOVA followed by Dunnett’s post hoc test.


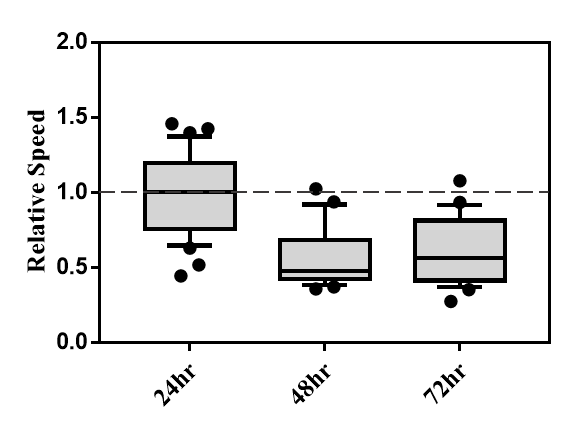


**Supplementary Figure S7.** Electrotaxis of animals exposed to *S. venezuelae* and *B. thuringiensis.* Refer to Figure 1 for a description of box plot. Worms fed with *E. coli* OP50 on NGM plates and MYM plates acted as controls for *S. venezuelae* fed animals. Likewise, for *B. thuringiensis*, *E. coli* OP50 on LB plate was used as a control. No significant change in *S. venezuelae* (p = 0.3242) and *B. thuringiensis* (p = 0.8320) fed animals was observed. The numbers of animals examined were: *E. coli* OP50 n = 26, *E. coli* OP50+MYM agar n = 22, *S. venezuelae* n= 27, *B. Thuringiensis* n = 24. Data was analyzed using one-way ANOVA followed by Dunnett’s post hoc test.


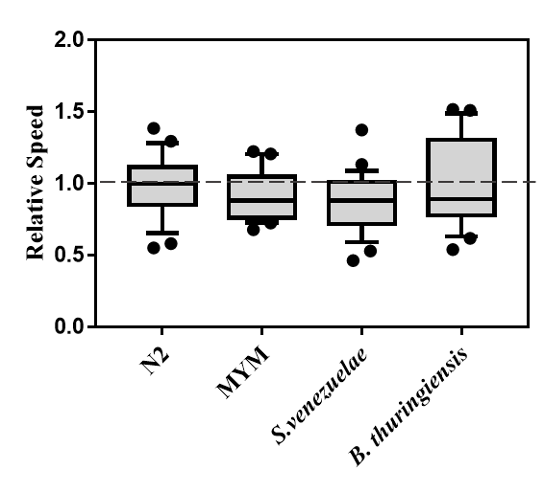


**Supplementary Figure S8.** Electrotaxis of wild-type animals fed with different microorganisms. Refer to Figure 1 for a description of box plot**.** No significant difference was observed between animals cultured on *E. coli* HB101 (p = 0.999), *E. coli* HT115 (p = 0.6587), *E. coli* DH5a (p = 0.9930), *C. aquaticus* DA1877 (p = 0.999), *A. tumefaciens* GV3101 (p = 0.121), or *E. carotovora* SCC3193 (p = 0.9511). The numbers of animals examined were: *E. coli* OP50 n = 20, *E. coli* HB101 n = 25, *E. coli* HT115 n = 16, *E. coli* DH5a n = 25, *C. aquaticus* DA1877 n = 30, *A. tumefaciens* GV3101 n = 25, and *P. carotovorum*  SCC3193 n = 19. Data was analyzed using one-way ANOVA followed by Dunnett’s post hoc test.


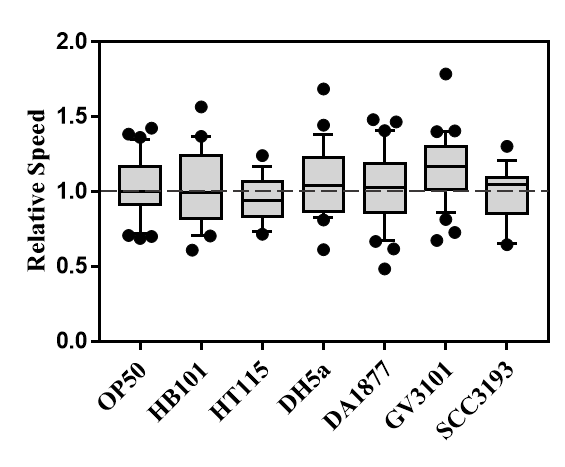


**Supplementary Figure S9.** Quantification of dopaminergic neurons in day-6 adults following exercise by shaking. The *dat-1::YFP* reporter was used to visualize neurons. No significant difference in neurodegeneration was observed (p = 0.7263, unpaired Student’s t-test). **F)** Control n = 48, Exercise n = 47. Data was analyzed using unpaired Student’s t-test.

**
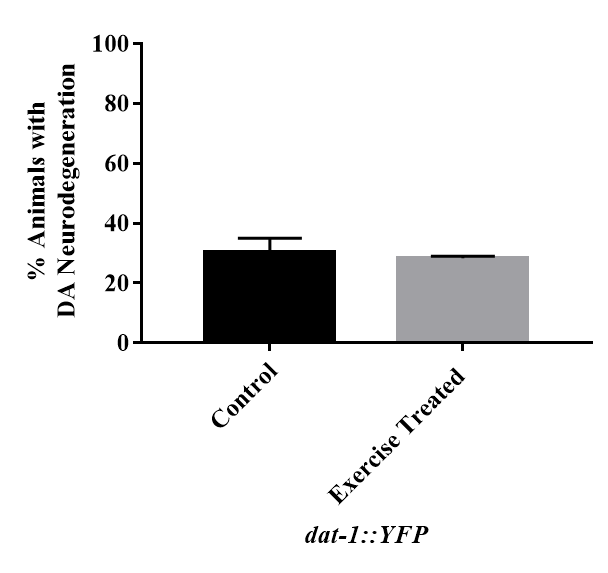
**
